# A Catalogue of Orthogonal Complementary Ligand Pairings for Palladium(II) Complexes

**DOI:** 10.1002/asia.202200272

**Published:** 2022-04-13

**Authors:** Jason S. Buchanan, Dan Preston

**Affiliations:** ^1^ Research School of Chemistry Australian National University Canberra ACT 2600 Australia

**Keywords:** palladium(II), self-sorting, complementarity, hydrogen bonding, systems chemistry

## Abstract

Molecular recognition is a form of information transfer, seen in the base pairing in DNA which is derived from the identity (acceptor or donor) and number of hydrogen bond sites within each base. Here we report *bis*‐ligand palladium(II) complexes that exhibit similar complementarity. Pd(II) has square planar four‐coordinate geometry, giving control over ligand orientation and denticity. Pairings were developed using ligand denticity (3 : 1 or 2 : 2), and hydrogen bond capability (AA:DD or AD:DA) or lack thereof. Five pairings were investigated, with two sets of four being found fully orthogonal. The two 3 : 1 pairings exhibited limited ligand exchange. The extent of this exchange varied dependant on solvent from 2 : 1 (desired to undesired) to 6 : 1. A reliable and varied set of ligand pairs have therefore been developed for *bis*‐ligand coordination sphere engineering in pursuit of sorting for complex molecular architectures and molecular‐level information storage and transfer events.

## Graphical abstract

**A catalogue of bis‐ligand Pd(II)** complexes has been developed. Each pair of ligands exhibits complementary to each other through denticity (2 : 2 or 3 : 1) and hydrogen bonding donor/acceptor capability. High levels of orthogonality between these pairings were demonstrated, with pair‐based sorting observed.

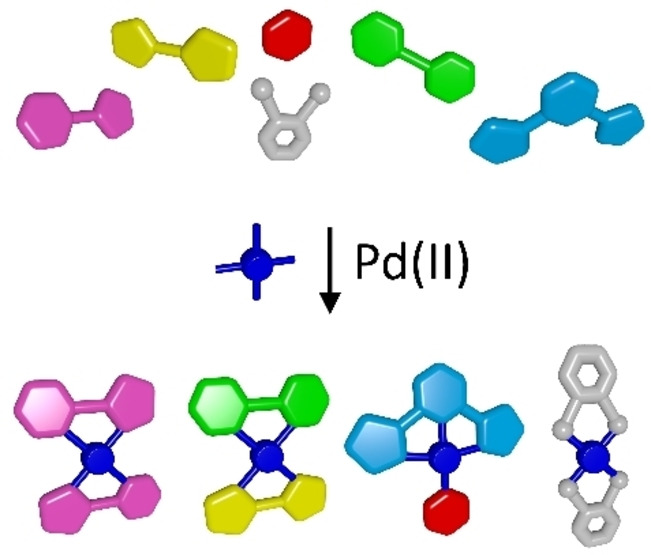

## Introduction

**Figure 1:**
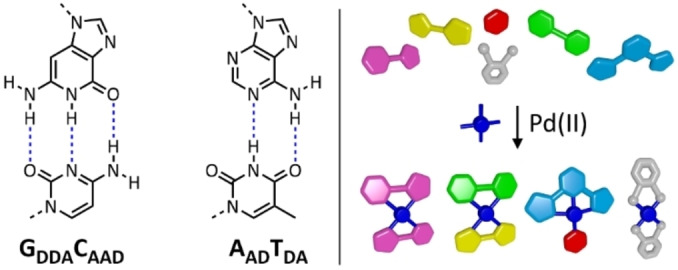
*Left*: the two base pairs that constitute DNA, which exhibit complementarity based upon the number and identity of their hydrogen bonding donor (D) and acceptor (A) sites. *Right*: the strategy adopted for orthogonal sorting between complementary ligand pairs in this project, based upon denticity and hydrogen bonding capacity.

Orthogonality in pairs of nucleic acids is central to their role in information storage, replication and transcription events. This is driven by complementarity in terms of the number and identity of hydrogen bond acceptor (A)/donor (D) sites. Two sets of base pairs (Figure 1 *left*) exist in nature, with guanine having a triple DDA array, cytosine having a triple AAD array, adenine a double AD array and thymine a double DA array. With a view towards expanding the potential for information storage, additional orthogonal artificial base pairs such as those seen in Hachimoji DNA have also been developed.^[1]^ Complementary hydrogen bonding arrays have also been used in synthetic tectons to engineer crystalline materials,^[2]^ and in the formation of discrete assemblies and receptors.^[3]^

Metal ions have been incorporated into DNA‐based and related structures.^[4]^ Control over the composition of the coordination sphere of metal ions has also been a topic of interest for chemists seeking to develop complex abiotic systems. For this to be effective in giving chemists control over product distribution outcome, orthogonality between different potential arrangements must be achieved. For example, Lehn and co‐workers have combined different amines, aldehydes and metal ions together in a single mixture and achieved parallel sorting of resultant imine‐based complexes, dependent on the preferred coordination geometry and number of the respective metal ions.^[5]^ Schmittel and co‐workers have also reported systems based around low symmetry ligands with varied binding sites and multiple metal ions for the formation of low symmetry macrocycles.^[6]^

Palladium(II) has proven to be a popular metal ion for the formation of polynuclear systems, due to its reliable square planar coordination geometry and behaviour, and hemi‐lability allowing for error correction in assembly processes.^[7]^ Considerable effort^[8]^ has gone into the development of multi‐component assemblies,^[9]^ including through ‘coordination sphere engineering’.^[10]^ Of particular relevance here are examples in which two ligands come together at a single metal ion:^[11]^ in these cases the metal ion acts as a node bringing together two sites, which must exhibit complementarity towards each other. This complementarity could plausibly arise from: 1) Denticity. For a square planar metal ion such as Pd(II), a tridentate and monodentate ligand will be complementary towards one another,^[12]^ as will two bidentate ligands towards each other.^[13]^ A 3 : 1 complex will by necessity be heteroleptic, while a 2 : 2 complex may be either heteroleptic or homoleptic. 2) Hydrogen bonding. In the case of *bis*‐bidentate systems, both the quantitative formation of heteroleptic complexes or control over isomer distribution for homoleptic complexes with lower symmetry ligands (i. e. whether they form head‐to‐head or head‐to‐tail) requires judicious ligand design. This has been accomplished with hydrogen bonding.^[14]^ Low symmetry ligands with a hydrogen bond acceptor and donor on opposite sides of the bidentate site have been demonstrated to form homoleptic *bis*‐complexes with square planar metal ions in a head‐to‐tail fashion.^[15]^ We have also recently reported heteroleptic *bis*‐bidentate coordination between a ligand with two acceptors and another ligand with two donors.^[12e]^

Presumably a similar approach could be adopted with 3 : 1 systems to allow sorting between different tridentate and monodentate ligands based around the identity of their hydrogen‐bonding components

Our goal in this project was to synthesise a series of *bis*‐ligand Pd(II) complexes which varied in their denticity and their hydrogen bonding capacity. It was our hope that not only would these complexes form in a controllable manner, but that subsequent pairings between them would be orthogonal to one another: in other words that the mixing of different complexes together (or indeed their *in situ* formation) would not result in ligand exchange (mismatching of the ligands) constituting the complexes (Figure 1 *right*). We hoped in this fashion to obtain a catalogue of different and orthogonal complementary ligand pairings that can be used with square planar metal ions.

## Results and Discussion

### Ligand synthesis, complex rationale and compound nomenclature

**Figure 2:**
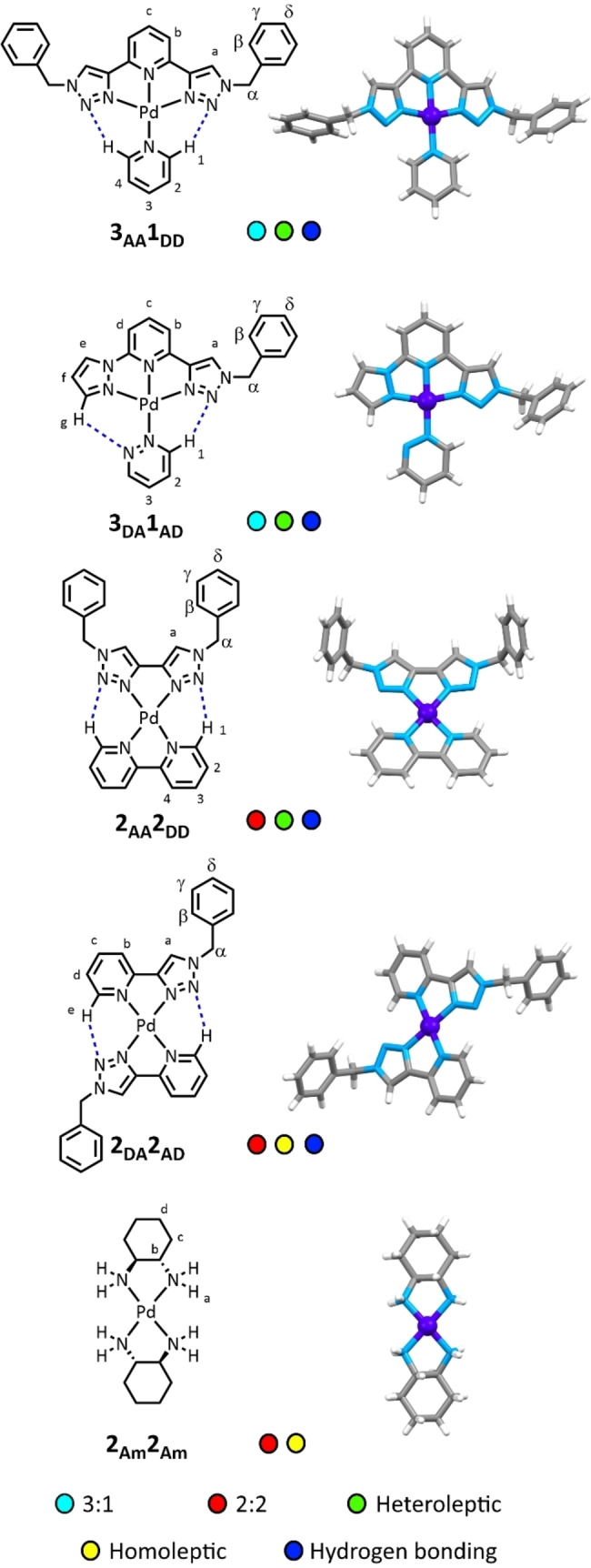
Chemical structures of complexes synthesised in this project, together with representations of X‐ray crystal structures. Crystal structures for **2_AD_2_DA_** and **2_Am_2_Am_** previously reported.[^15d^, ^19^] Colours: carbon grey, hydrogen white, nitrogen light blue, palladium dark blue. Counterions, and any solvent or co‐crystallisation agents omitted for clarity.

Ligands were named through a number denoting their denticity (**3** for tridentate, **2** for bidentate or **1** for monodentate), and a subscript informing as to their character. This character could relate to their hydrogen bonding capabilities in the position *ortho* to their coordinating nitrogen(s), either a donor (_**D**_) C−H unit or an acceptor (_**A**_) nitrogen atom. For example, monodentate pyridine with two *ortho* C−H units is denoted **1_DD_** (Figure 2). Hydrogen bonding was not critical for bidentate (1S,2S)‐(+)−1,2,‐diamonohexane within this particular system, and so this ligand is subscripted for its amino coordinating groups: **2_Am_**.

Each ligand was chosen or selected for its capacity to either be complementary to another ligand, or exhibit self‐complementarity. For example, **3_AA_** exhibits potential complementarity to **1_DD_** in terms of both denticity and hydrogen bonding capability for the formation of a heteroleptic complex (Figure 2). Other complementary pairs were **3_AD_** to **1_DA_**, and **2_AA_** to **2_DD_**. For homoleptic complex with low symmetry ligands **2_DA_**, self‐complementarity in the formation of head‐to‐tail *bis*‐complexes with square planar metal ions due to hydrogen bonding has previously been observed. The complementarity between these ligands was designed not only through targeting favourable interactions, but also through avoidance of unfavourable ones. For example, in the planned combination of **2_AA_** and **2_DD_**, *bis*‐homoleptic complexations would result in the *ortho* protons H_1_ of **2_DD_** being directed at each other in a sterically unfavourable manner, while in **2_AA_**, the lone pairs of the triazole N2 nitrogen atoms would likewise be unfavourably aligned.

Lastly, **2_Am_** was hoped to form a *bis*‐complex in an orthogonal fashion to other complexes through the absence of favourable steric or hydrogen bonding considerations. While the **2_Am_** ligand has amino hydrogen atoms available for hydrogen bonding, initial modelling^[16]^ suggested that these would be out of the plane of the lone pair of electrons on ligands with acceptor atoms, and that any hydrogen bonds would be at more acute (and therefore less favourable) angles.

Furthermore, **2_Am_** is not significantly sterically bulky: the related ethylenediamine ligand has previously been used by Chand and co‐workers in the formation of heteroleptic Pd(II) complexes for this reason.^[14b]^

Complexes are named through conjunction of the two ligands present: a [Pd(L)(L’)]^2+^ complex from **3_AA_** and **1_DD_** would be named **3_AA_1_DD_**, while the *bis‐*bidentate complex from **2_DA_** (for example) would be **2_DA_2_AD_**. Ligands were either commercially available, or synthesised according to literature procedures for **3_AA_**,^[17]^ **2_AA_**,^[18]^ and **2_DA_**.^[15d]^ The one novel ligand, **3_DA_**, was synthesised through CuAAC “click” chemistry and characterised (Supporting Information) through nuclear magnetic resonance (NMR) spectroscopies and high resolution electrospray ionisation mass spectrometry (HR‐ESI‐MS).

### Complex synthesis and characterisation

All complexations were carried out with [Pd(CH_3_CN)_4_](BF_4_)_2_. Pd(II) was chosen not only for its square planar coordination geometry, but for ease of synthesis due to hemi‐lability (in comparison to, for example, Pt(II)). Homoleptic complexes were formed from the 2 : 1 combination of ligand to metal ion, while heteroleptic complexes were formed from the 1 : 1 : 1 combination of the first and second ligand and metal ion. Complexations could be carried out with the same results in a variety of solvents: [D_6_]DMSO, [D_3_]acetonitrile, and [D_6_]acetone and their non‐deuterated equivalents. The complexes were all isolated through vapour diffusion of diethyl ether into a DMSO/acetonitrile solution of the applicable complex in good yields (71–83%). The complex **2_DA_2_AD_** has been previously reported and characterised.^[15d]^ The complex **2_Am_2_Am_** has previously been synthesised as the tetracyanoplatinate salt.^[19]^ The ligand **2_Am_** could not be successfully complexed in acetone due to competing imine bond formation.

HR‐ESI‐MS in DMSO/acetonitrile confirmed complex formation. For example, the homoleptic complex **2_Am_2_Am_** showed +1 peaks at 333.1277 and 420.1406 *m/z*, corresponding to the [Pd(**2_Am_**)_2_−H]^+^ and [Pd(**2_Am_**)_2_+BF_4_]^+^ species respectively. In the heteroleptic complex **3_AA_1_DD_**, there was a +1 peak at 665.1213 *m/z* from the [Pd(**3_AA_**)(**1_DD_**)+BF_4_]^+^ species, and also at 487.0636 *m/z*. This second peak is derived from loss of the benzyl group from the complex and formation of a triazolato group on the ligand, i. e. [Pd(**3_AA_**−bz)(**1_DD_**)]^+^. This fragmentation was commonly observed with the portion of this set of complexes with pyridyl‐triazole functionality and has previously been observed in related complexes through mass spectrometry^[20]^ and through synthetic design.^[21]^ Lastly, the 3 : 1 complexes also showed some peaks from species in which the monodentate ligand was displaced under mass spectrum conditions by an anion readily available in the spectrometer (hydride and fluoride). The other MS results likewise confirmed the formation of *bis*‐ligand complexes in a similar fashion, including with fluoride counterions, which presumably originate from the fragmentation of tetrafluoroborate counterions (Supporting Information).

**Figure 3:**
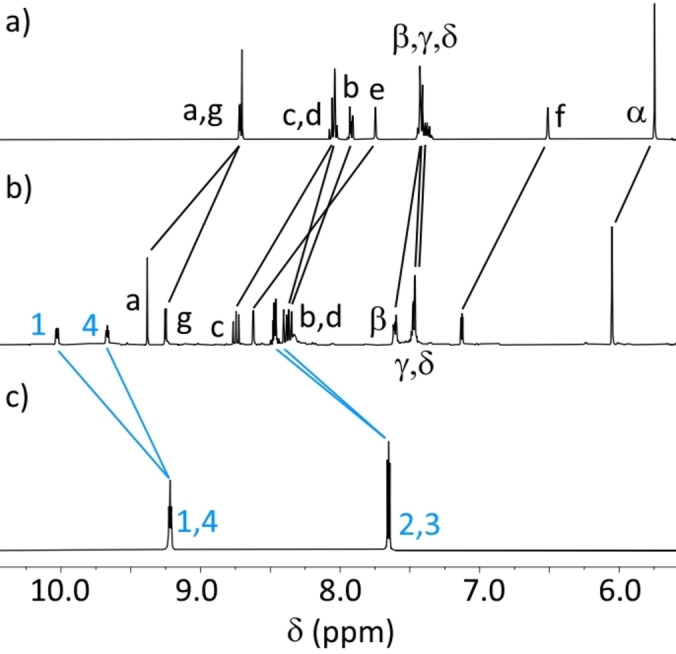
Partial ^1^H NMR spectra (400 MHz, [D_6_]acetone, 298 K) of a) **3_AD_**, b) **3_AD_1_DA_**, and c) **1_DA_**.

^1^H NMR spectroscopy gave good evidence of complex formation. Downfield shifting of chemical resonances in comparison to the ‘free’ ligands was observed, indicative of complexation (for example for **3_AD_1_DA_** see Figure 3, for other complexes see Supporting Information). In each case, a single set of peaks per ligand environment was observed. In conjunction with the MS results, this indicates clean formation of heteroleptic complexes, or the formation of a single isomer in the case of homoleptic complexes with low symmetry ligands. In ‘free’ **1_DA_**, there are only two proton resonances. Upon complexation for **3_AD_1_DA_**, the pyridazine resonances split from two to four environments (Figure 3b and c), consistent with coordination to a single **1_DA_** nitrogen on the ^1^H NMR time scale. For **2_AA_2_DD_**, ^1^H NOESY NMR spectroscopy showed through‐space couplings between the *ortho* proton 1 of **2_DD_** and the methylene proton α of **2_AA_**, indicating formation of the heteroleptic complex (Supporting Information).

**Figure 4:**
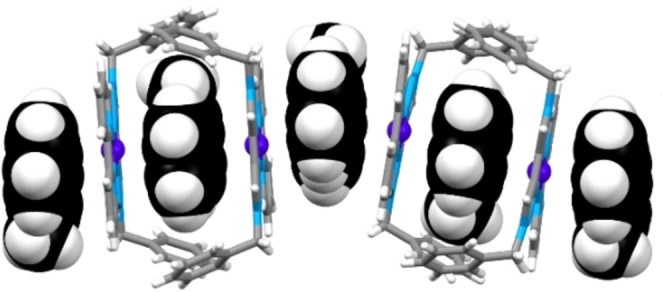
Representation of the alternating **3_AD_1_DA_** and 9‐methylanthracene columnar stacks observed in the X‐ray crystal structure from their co‐crystallisation. Complex shown in tube form, 9‐methylanthracene in spacefilling form. Counterions and other 9‐methylanthracene molecules omitted for clarity. Colours: carbon grey in complex, black in 9‐methylanthracene, hydrogen white, nitrogen light blue, palladium dark blue.

X‐ray crystallography was also employed for proof of structures (Figure 2). **2_Am_2_Am_** has previously been crystallographically characterised, albeit as the tetracyanoplatinate salt.^[19]^ The X‐ray structure of **2_DA_2_AD_** has also been reported^[15d]^ and the structure reveals expected formation of the head to tail isomer. Three of the novel complexes were successfully crystallised: **3_AA_1_DD_** (P2_1_/c, *R*_1_=3.4%), **3_AD_1_DA_** (P2_1_/n, *R*_1_=2.5%), and **2_AA_2_DD_** (P2_1_/c, *R*_1_=7.2%). For **3_AA_1_DD_** and **2_AA_2_DD_**, these were successfully obtained using anthracene or 9‐methylanthracene as co‐crystallisation agents. The affinity between electron rich neutral aromatics and planar cationic complexes^[22]^ has previously been employed in our group for this purpose.^[20]^ The co‐crystallised structures revealed columnar stacks of alternating aromatic agent and the specific complex (for example for **3_AD_1_DA_** and 9‐methylanthracene, Figure 4, also see Supporting Information). The data from the X‐ray crystallography were all consistent with NMR and MS data in supporting formation of the targeted complexes. Donor‐acceptor distances (C−H−N) for hydrogen bonds in the applicable complexes (**3_AA_1_DD_**, **3_AD_1_DA_**, **2_AA_2_DD_** and **2_DA_2_AD_**) ranged from 2.301–2.958 Å, indicating that the targeted hydrogen bonding arrays in these complexes were feasible (Table 1).

**Table 1:** Intramolecular hydrogen bond distances in studied complexes. All complexes had one single chemically independent hydrogen bond, except for **3_AD_1_DA_** which has two. The size of the downfield shift in the ^1^H NMR spectrum upon complexation is also given for proton resonances involved in hydrogen bonds.

Compound	D(N−H−C) [Å]	Δδ [ppm]^[c]^
**3_AA_1_DD_**	2.958^[a]^	0.41 (H_1_)
**3_AD_1_DA_**	2.360, 2493	0.52 (H_1_), 0.61 (H_g_)
**2_AA_2_DD_**	2.286^[a]^	1.05 (H_1_)
**2_DA_2_AD_**	2.301^[b]^	0.85 (H_e_)

[a] Chemically equivalent but crystallographically inequivalent hydrogen bond distances given as averages. [b] See Ref. [23]. [c] In [D_6_]DMSO, 400 MHz, 298 K, expressed as downfield shift.

In particular, the structure of **3_AD_1_DA_** was particularly convincing as to the potential for hydrogen bonding. Despite the capacity for the monodentate ligand in 3 : 1 complexes to rotate out of the square planar plane, in this complex **1_DA_** is close to coplanar (2.14(8)° out of plane). Rotation could potentially result in a less sterically encumbered conformation, but instead the planarity of the complex, together with the directionality of hydrogen bond acceptors and donors present, strongly suggests that hydrogen bonding can significantly contribute to complex conformation.

### Combinatorial studies

**Figure 5:**
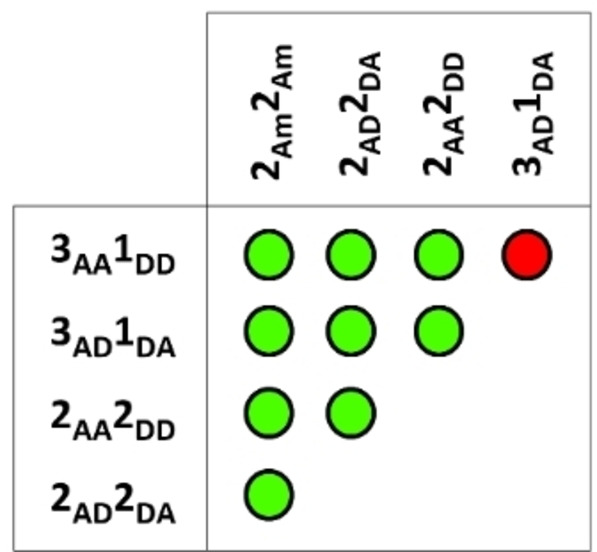
Results from the 1 : 1 combination of all of the complexes in this study. Green circles indicate no ligand exchange, while red circles indicate that ligand exchange occurs.

With five complexes in hand, combinatorial studies were now carried out to investigate whether exchange between ligand pairs would occur. Each complex was combined in turn in a 1 : 1 fashion, in a variety of deuterated solvents. Again, [D_6_]acetone could not be used with the **2_Am_** ligand, due to complications with imine formation. For the majority of the 1 : 1 combinations, no mixing was seen regardless of the solvent. The exception was the combination of **3_AA_1_DD_** and **3_AD_1_DA_**, for which exchange was observed (Figure 5).

While the combination of **3_AA_1_DD_** and **3_AD_1_DA_** in a variety of solvents (deuterated DMSO, acetone, acetonitrile and nitromethane) resulted in spectra with some to many coincident peaks, inspection revealed the existence of four separate species (Supporting Information). Unfortunately, because products and mismatched complexes only differed by 1 atomic mass unit, MS data was not useful. To identify the extra peaks, the mismatched complexes **3_AA_1_DA_** and **3_AD_1_DD_** were independently and separately prepared (Supporting Information). Overlaid ^1^H NMR spectra ([D_6_]DMSO) for the four complexes clearly revealed that they made up the components present within the equilibrium mixture (Supporting Information). Density functional theory (DFT) calculations (B86, def2‐TZVP, def2/J, DMSO solvent field) were carried out on the four complexes, and the energies were compared. Between mismatched reactants and products there was very little energetic difference from the computations (1.6 kJ mol^−1^), confirming the central position of the equilibrium.

**Figure 6:**
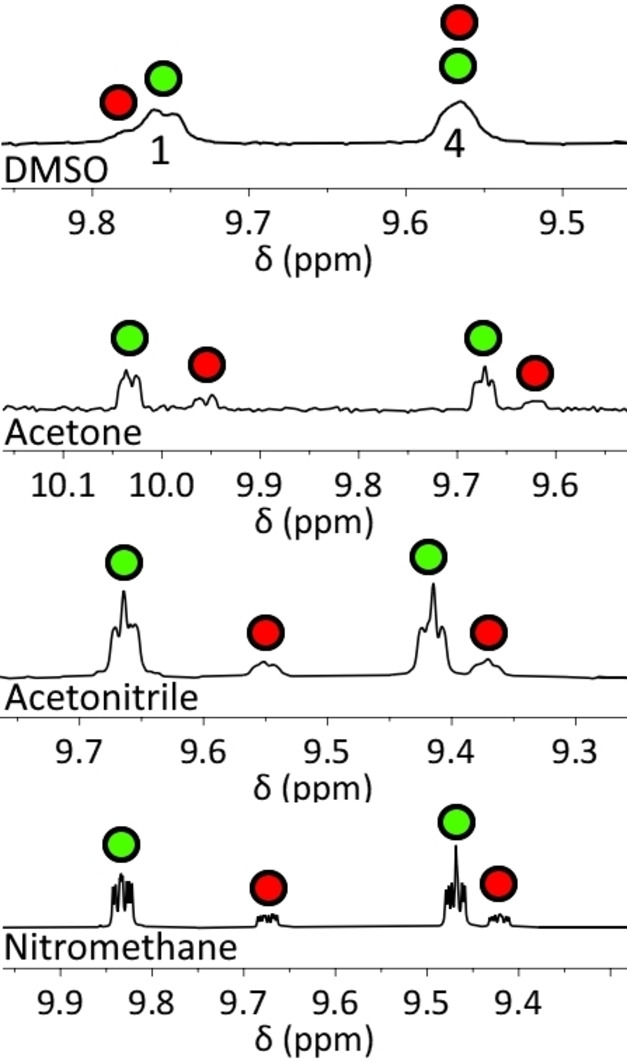
Partial ^1^H NMR spectra (400 MHz, 298 K) from the 1 : 1 combination of **3_AA_1_DD_** and **3_AD_1_DA_**, in deuterated DMSO, acetone, acetonitrile and nitromethane, showing the **1_DD_** pyridazine peaks H_1_ and H_4_. These peaks are present in ‘unscrambled’ **3_AD_1_DA_** (green circles) and ‘scrambled’ **3_AA_1_DA_** (red circles).

From the ^1^H NMR spectra, the proportion of the species present was not statistical: the targeted complexes predominated, i. e. the ‘unscrambled’ **3_AA_1_DD_** and **3_AD_1_DA_** complexes were more favoured than the ‘scrambled’ **3_AA_1_DA_** and **3_AD_1_DA_** complexes. The degree of predomination depended on the solvent used (Figure 6).

Unsurprisingly, this can be correlated to the hydrogen bonding capabilities of these solvents. All are poor hydrogen bond donors, but all can accept hydrogen bonds to a greater or lesser extent. Using the hydrogen bond acceptor parameter β,^[24]^ in strongly hydrogen bonding DMSO (β(sulfoxide)=8.9), the exchanged/non‐exchanged ratio=2 : 1, and K_eq_=4. In moderately hydrogen bond accepting acetone (β(ketone)=5.8), the ratio is 3 : 1 and K_eq_ is ≈10. In the more poorly hydrogen bond accepting solvents acetonitrile (β(nitrile)=4.7) and nitromethane (β(nitroalkane)=3.7), the ratio was ≈6 : 1 and K_eq_=20–30. From these data we infer that 3 : 1 complexes are more prone to mixing than 2 : 2 analogues, and that this is likely due to competitive hydrogen bond accepting from solvent. The ability of the tridentate and monodentate ligands to rotate with respect to each other, which is not present in 2 : 2 complexes, is likely a significant contributing factor here: it allows solvent access for hydrogen bonding, as well as relief of any steric strain in mismatched complexes.

**Figure 7:**
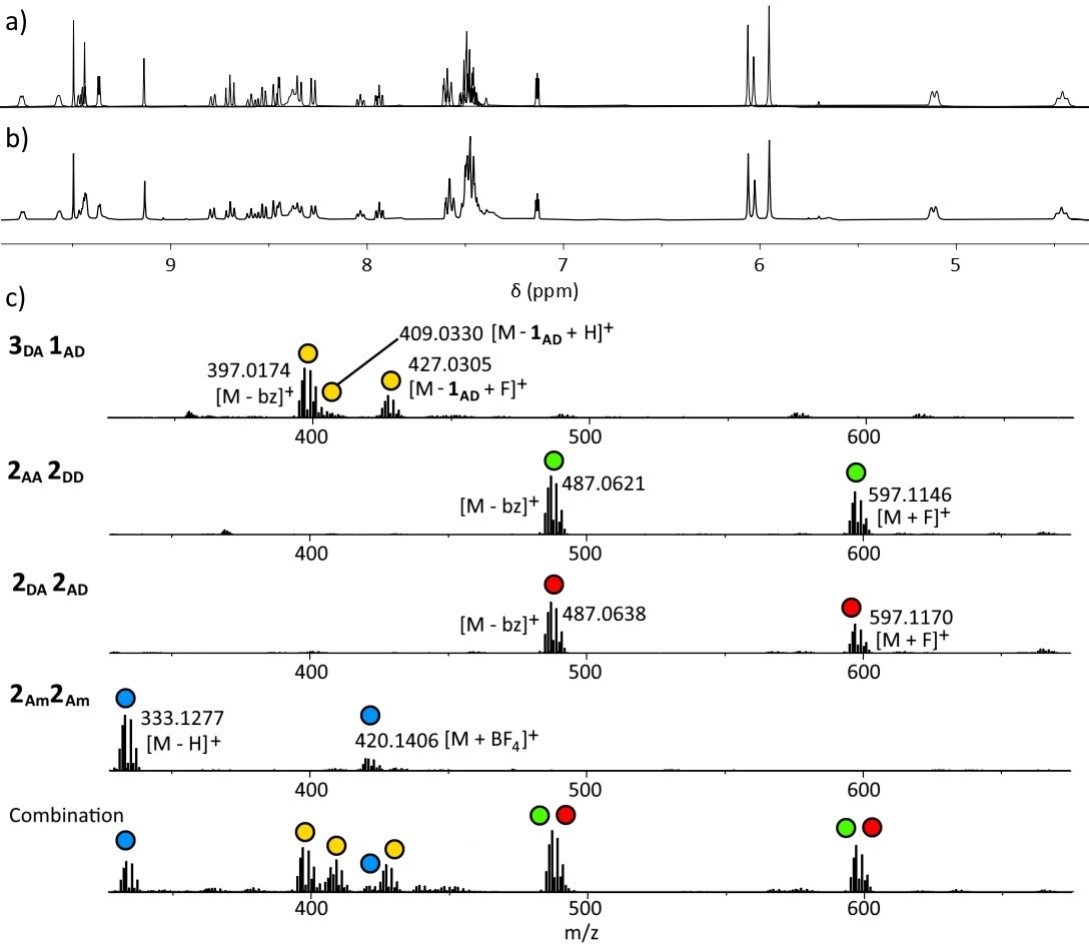
Partial ^1^H NMR spectra (400 MHz, [D_6_]DMSO, 298 K) spectra of a) overlaid four spectra of individual complexes **3_AD_1_DA_**, **2_AA_2_DD_**, **2_DA_2_AD_**, and **2_Am_2_Am_**, and b) the spectrum of the combination of the four complexes. Below is c) partial mass spectra (DMSO/acetonitrile) for individual complexes **3_AD_1_DA_**, **2_AA_2_DD_**, **2_DA_2_AD_**, and **2_Am_2_Am_**, and their combination.

The capacity for multiple complexes to be combined without mismatching was now explored. Equimolar combinations of all three *bis*‐bidentate complexes with either of the 3 : 1 complexes were now carried out in [D_6_]DMSO. ESI‐MS on the mixtures showed conclusively that mismatching had not occurred: no peaks due to exchanged ligand combinations were observed (Figure 7c and Supporting Information). The cationic portion of several complexes (**3_AA_1_DD_**, **2_AA_2_DD_**, **2_DA_2_AD_**) are identical in molecular weight and showed similar speciation, but it must be noted that scrambling would result in complex formation that diverged from these molecular formulae. This was not observed. The loss of a single benzyl group and formation of the triazalato ligand (*see above*) was again a predominant theme. As with HR‐ESI‐MS of individual 3 : 1 complexes, some speciation indicative of [Pd(tridentate)(anion)]^+^ was observed where anion=hydride, fluoride, methoxide, formate, but again no mismatching was discernible, suggesting fragmentation rather than exchange. These data were corroborated by the ^1^H NMR spectra (Figure 7a and b and Supporting Information) which for the mixtures were an excellent match for the overlaid spectra of (in both cases) the four individual complexes. Via ^1^H NMR spectroscopy, no evidence for mismatching between either of the four sets of complexes was observed.

To confirm that the observed sorting was occurring under thermodynamic control, **2_AA_**, **2_DD_**, **2_DA_**, **2_Am_**, **3_AD_** and **1_DA_**, were combined in a 1 : 1 : 2 : 2 : 1 : 1 ratio. To this was added four equivalents of Pd(II). The ^1^H NMR spectrum (Supporting Information) thus obtained was essentially indistinguishable from the spectrum from the direct combination of pre‐formed complexes. This indicates that the orthogonality observed between the pairings involved in this study is thermodynamic rather than kinetic in nature, and up to four separate pairs can self‐assemble without exchange.

## Conclusion

A series of five complementary ligand pairings were investigated, all based around coordination to Pd(II). These were complementary towards one another in terms of denticity (3 : 1 or 2 : 2) and the identity of hydrogen bonding sites (AA:DD, DA:AD) *ortho* to coordinating nitrogen atoms, or in the case of ligand **2_Am_**, was ‘non‐complementary’ towards any other ligand.

All targeted 2 : 2 complexes were orthogonal to each other, and to either of the 3 : 1 complexes. The two 3 : 1 complexes exhibited limited ‘mismatching’, likely due to rotational capabilities around the palladium‐monodentate ligand bond in 3 : 1 complexes not present in 2 : 2 analogues. The degree of this mismatching depended on the hydrogen bond acceptor capabilities of the solvent and ranged from a 2 : 1 ‘matched’ to ‘mismatched’ ratio for DMSO to ≈6 : 1 for acetonitrile or nitromethane.

Quadruple complex combinations were carried out between the three *bis*‐bidentate complexes and either of the 3 : 1 complexes, without observation of mismatching. These confirmed the orthogonality of the ligand pairings employed. Therefore, a catalogue of up to four orthogonal pairings was developed. Even in the fifth combination between the two 3 : 1 complexes, in the correct solvent the targeted pairing was reasonably well preferred: a 6 : 1 ratio still represents an unprecedented level of narcissistic sorting in 3 : 1 mononuclear Pd(II) complexes. Unlike previous efforts, this has been accomplished with a single metal ion with a single coordination number/geometry preference.

As stated in the introduction, the formation of lower symmetry/higher complexity abiotic structures is at the forefront of the minds of chemists seeking to develop systems capable of emulating the power and breadth of natural molecular machinery. In a broader sense, complementarity pairings lie at the heart of molecular storage and transfer events. A catalogue of reliable pairings of ligands with square planar metal ions can only advance these aims. We are currently developing self‐replicating molecules based on the pairings we have reported here.

## Conflict of interest

The authors declare no conflict of interest.

1

## Supporting information

As a service to our authors and readers, this journal provides supporting information supplied by the authors. Such materials are peer reviewed and may be re‐organized for online delivery, but are not copy‐edited or typeset. Technical support issues arising from supporting information (other than missing files) should be addressed to the authors.

Supporting Information

## Data Availability

The data that support the findings of this study are available in the supplementary material of this article.
