# Supplementary material for: A Catalogue of Orthogonal Complementary Ligand Pairings for Palladium(II) Complexes
Source: Chem Asian J. 2022 Apr 13;17(11):e202200272. doi: 10.1002/asia.202200272 (PMC9324840; doi:10.1002/asia.202200272)
Supplement: Supplementary file 1 — Supporting Information [file ASIA-17-0-s001.pdf]

CHEMISTRY
AN ASIAN JOURNAL

Supporting Information

A Catalogue of Orthogonal Complementary Ligand Pairings for Palladium(II) Complexes

Jason S. Buchanan and Dan Preston*© 2022 The Authors. Chemistry - An Asian Journal published by Wiley-VCH GmbH. This is an open access article under the terms of the Creative Commons Attribution License, which permits use, distribution and reproduction in any medium, provided the original work is properly cited.

1. Experimental

1.1. General

Unless otherwise stated, all reagents were purchased from commercial sources and used without further purification, except for:

Precursors

Benzyl azide[1]

2-(1H-pyrazol-1-yl)-6-((trimethylsilyl)ethynyl)pyridine[2]

Ligands

$\mathbf{2_{AA}}$[3]

$\mathbf{2_{DA}}$[4]

$\mathbf{3_{AA}}$[5]

Complexes

$\mathbf{2_{DA}2_{AD}}$[4]

which were synthesised according to literature procedures. Solvents were laboratory reagent grade. Petroleum ether (PE) refers to the fraction of petrol boiling in the range 40 – 60 °C, dichloromethane (DCM), ethylenediaminetetraacetate (EDTA), dimethyl sulfoxide (DMSO), dimethylformamide (DMF). ^{1}H and ^{13}C NMR spectra were recorded on either a 400 MHz Varian 400-MR, a Varian 500 MHz AR, a JEOL 400 MHz, a JEOL 600 MHz spectrometer, a Bruker Avance 400 MHz or a Bruker Avance 700 MHz spectrometer. Chemical shifts are reported in parts per million and referenced to residual solvent peaks ($CDCl_3$: ^{1}H δ 7.26 ppm, ^{13}C δ 77.16 ppm; [D_6]DMSO: ^{1}H δ 2.50 ppm; ^{13}C δ 39.52 ppm, [D_3]acetonitrile: ^{1}H δ 1.94; [D_3]nitromethane: ^{1}H δ 4.30 ppm; [D_6]acetone: ^{1}H δ 2.05 ppm, ^{13}C δ 29.9). Coupling constants (J) are reported in Hertz (Hz). Standard abbreviations indicating multiplicity were used as follows: m = multiplet, q = quartet, quin = quintet, t = triplet, dt = double triplet, d = doublet, dd = double doublet, s = singlet, br = broad. Electrospray mass spectra (HR ESI-MS) were collected on a Bruker micrOTOF-Q spectrometer or a Waters Synapt G2-S1 HDMS spectrometer.

CAUTION: WHILE NO PROBLEMS WERE ENCOUNTERED DURING THIS WORK, AZIDES ARE EXPLOSIVE AND CARE SHOULD BE TAKEN WHEN DEALING WITH THEM.

1.2. Ligand

1.2.1. 3_{DA}

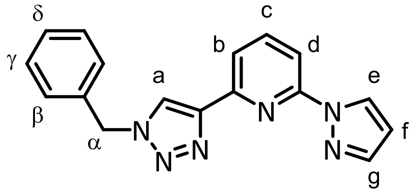

2-(1H-pyrazol-1-yl)-6-((trimethylsilyl)ethynyl)pyridine[2] (215 mg, 0.892 mmol) and sodium carbonate (157 mg, 1.47 mmol) were stirred in DMF (9 mL) for 15 minutes. To the dark brown stirring solution was added benzyl azide[1] (103 mg, 0.776 mmol), $CuSO_4{\cdot}5H_2O$ (94 mg, 0.38 mmol) and sodium ascorbate (151 mg, 0.760 mmol), followed by water (1 mL). The mixture was stirred at room temperature overnight. 0.1 M EDTA/NH_4OH aqueous solution (40 mL) was added to the dark yellow solution and stirred vigorously for a further 30 minutes before being left to rest for 10 minutes. The organic layer was washed with water (5 x 80 mL) and solvent removed under vacuum. Purification was performed through column chromatography on silica (DCM to 1:20 DCM/acetone) affording a pale brown product, 3_{DA} (203 mg, 0.671 mmol, 89%). ^1H NMR (400 MHz, [D$_6$]DMSO, 298 K) δ: 8.93 (1H, s, H_a), 8.81 (1H, d, J = 1.83 Hz, H_e), 8.07 (1H, t, J = 7.95 Hz, H_c), 7.95 (1H, d, J = 7.7 Hz, H_d), 7.84 (2H, m, $H_{b,g}$), 7.39 (5H, m, $H_{\beta,\gamma,\delta}$), 6.61 (1H, dd, J = 1.71 Hz, J = 0.98 Hz, H_f), 5.70 (2H, s, H_α). ^{13}C NMR (100 MHz, $CDCl_3$, 298 K) δ: 151.2, 148.8, 148.4, 142.2, 139.7, 134.6, 129.3, 129.0, 128.2, 127.0, 122.3, 117.8, 111.6, 107.8, 54.5. HR ESI-MS ($CDCl_3$/methanol) m/z = 325.1238 $[M + Na]^+$ (calc. for $C_{17}H_{14}N_6$, 325.1178).

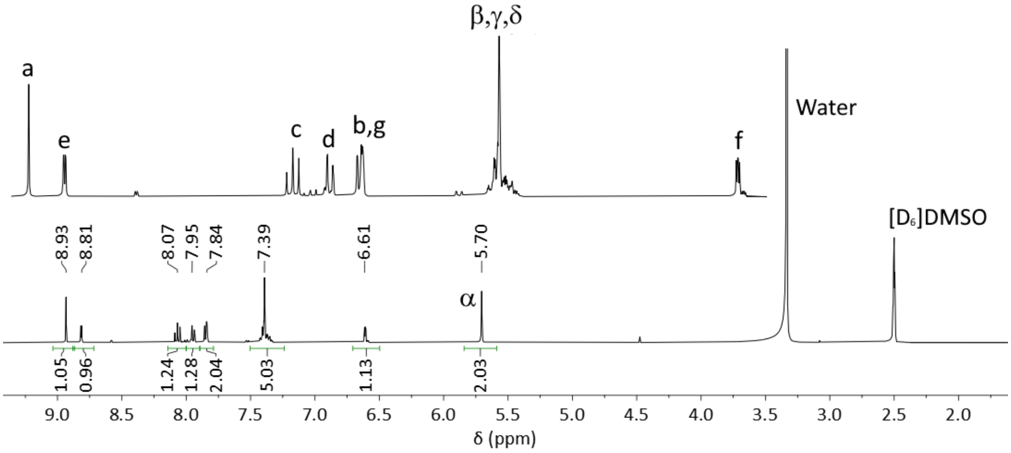

Figure 1.1 ^1H NMR spectrum (400 MHz, [D$_6$]DMSO, 298 K) of $\mathbf{3_{DA}}$.

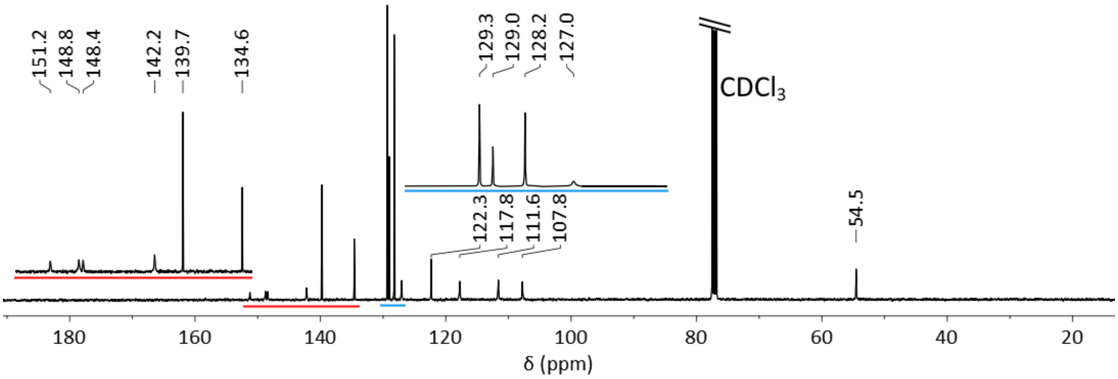

Figure 1.2 ^{13}C NMR spectrum (400 MHz, $CDCl_3$, 298 K) of $\mathbf{3_{DA}}$.

1.3. Complexes

1.3.1. General synthetic procedure

The following components were combined in 600 µL [D$_6$]DMSO: [Pd(CH_3CN)$_4$](BF_4)$_2$ (22.5 µmol) and two equivalents of ligand, i.e. for homoleptic complexes, 45 µmol of ligand, for heteroleptic complexes, 22.5 µmol of each ligand. For accuracy, these were added from previously prepared stock solutions. After confirmation of product formation through NMR spectroscopies, acetonitrile (2 mL) was added, and vapour diffusion of diethyl ether into the solution gave the products as crystalline solids. The previously reported **2$_{DA}$2$_{AD}$** complex[4] was not isolated. Exact masses and yields given below.

1.3.2. NMR studies

Using the ratios given above, NMR studies were carried out through combination of components in masses appropriate to the solubility of the complexes in the given solvent. Solubility was lowest in [D$_6$]acetone, and in the case of **2$_{AA}$2$_{DD}$**, a minimal amount of [D$_6$]DMSO was required to solubilise ligand **2$_{AA}$**. The complex **2$_{Am}$2$_{Am}$** was not able to be prepared in acetone, due to imine bond formation.

1.3.3. 2$_{AA}$2$_{DD}$

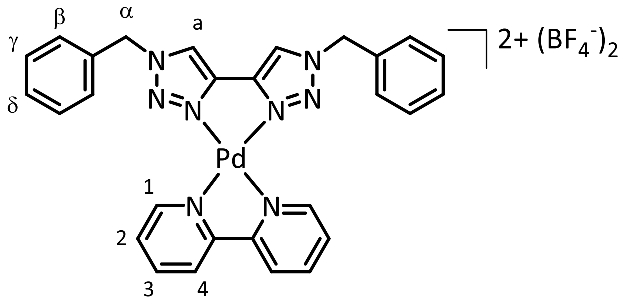

[Pd(CH_3CN)$_4$](BF_4)$_2$: 10.0 mg, 22.5 µmol. **2$_{AA}$**[3]: 7.13 mg, 22.5 µmol. **2$_{DD}$**: 3.52 mg, 22.5 µmol. Yield: 12.90 mg, 17.10 µmol, 76%. ^{1}H NMR (400 MHz, [D$_6$]Acetone, 298 K) *δ*: 9.69 (2H, d, *J* = 5.86, H$_1$), 9.12 (2H, s, H$_a$), 8.76 (2H, d, *J* = 8.19 Hz, H$_4$), 8.60 (2H, t, *J* = 7.95 Hz, H$_3$), 8.07 (2H, t, *J* = 5.99 Hz, H$_2$), 7.66 (2H, d, *J* = 6.24 Hz, H$_β$), 7.46 (2H, m, H$_{γ,δ}$), 6.11 (4H, s, H$_α$). ^{13}C NMR (100 MHz, [D$_6$]Acetone, 298 K) *δ*: 157.9, 152.9, 144.3, 139.9, 134.5, 130.2, 130.0, 129.9, 129.5, 126.3, 125.8, 57.4. HR ESI-MS (DMSO/Acetonitrile) *m/z* = 597.1146 [M + F]$^+$ (calc. for Pd($C_{10}H_8N_2$)($C_{18}H_{16}N_6$)F, 597.1153).

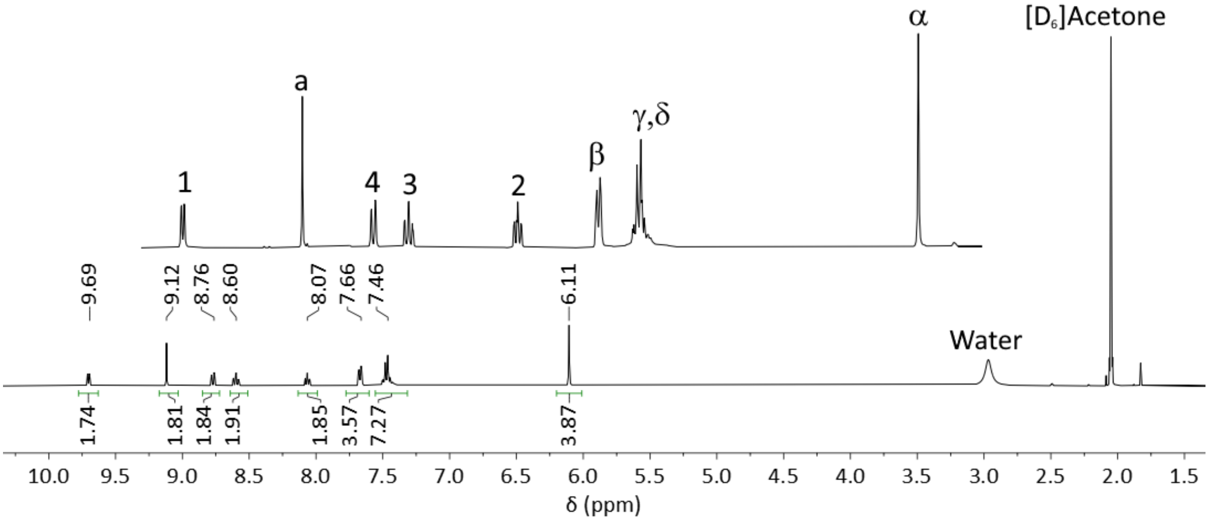

Figure 1.3 ^{1}H NMR spectrum (400 MHz, [D$_6$]Acetone, 298 K) of **2$_{AA}$2$_{DD}$**.

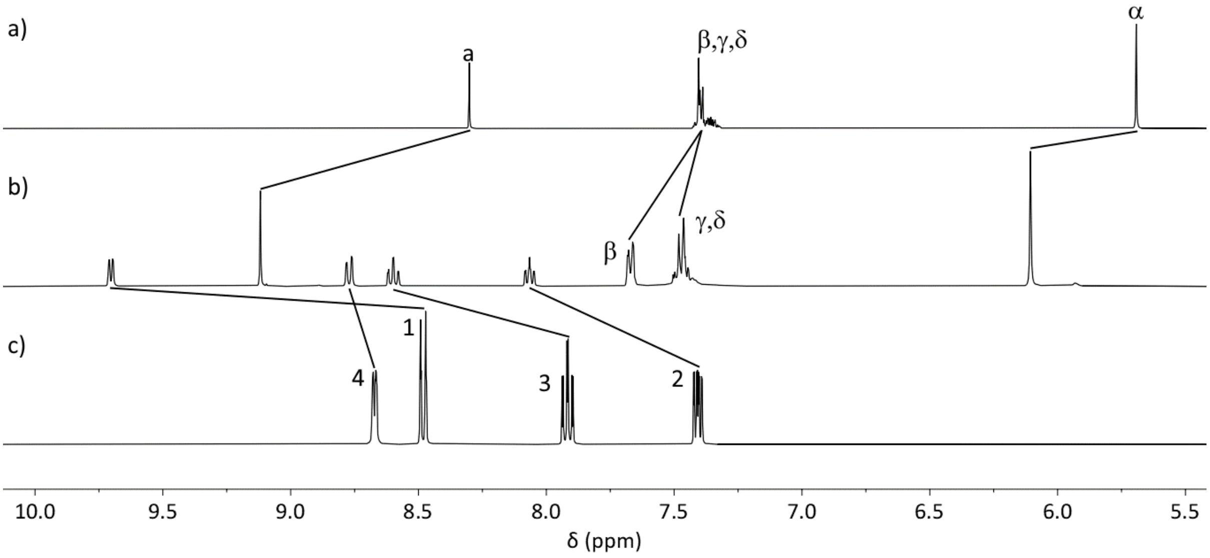

Figure 1.4 Stacked partial ^{1}H NMR spectra (400 MHz, [D$_6$]Acetone, 298 K) of a) $\mathbf{2_{AA}}$, b) $\mathbf{2_{AA}2_{DD}}$, and c) $\mathbf{2_{DD}}$.

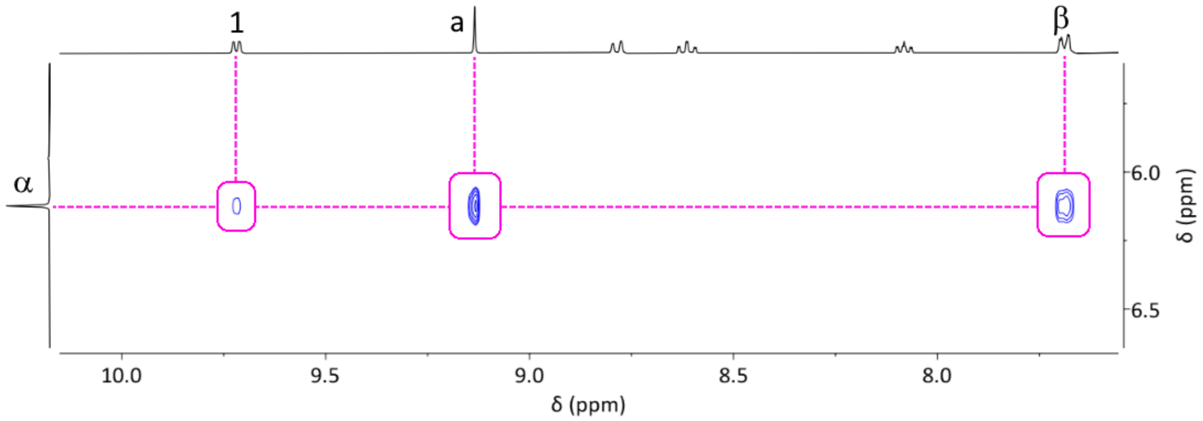

Figure 1.5 Partial ^{1}H NOESY NMR spectrum spectra (400 MHz, [D$_6$]DMSO, 298 K) of $\mathbf{2_{AA}2_{DD}}$.

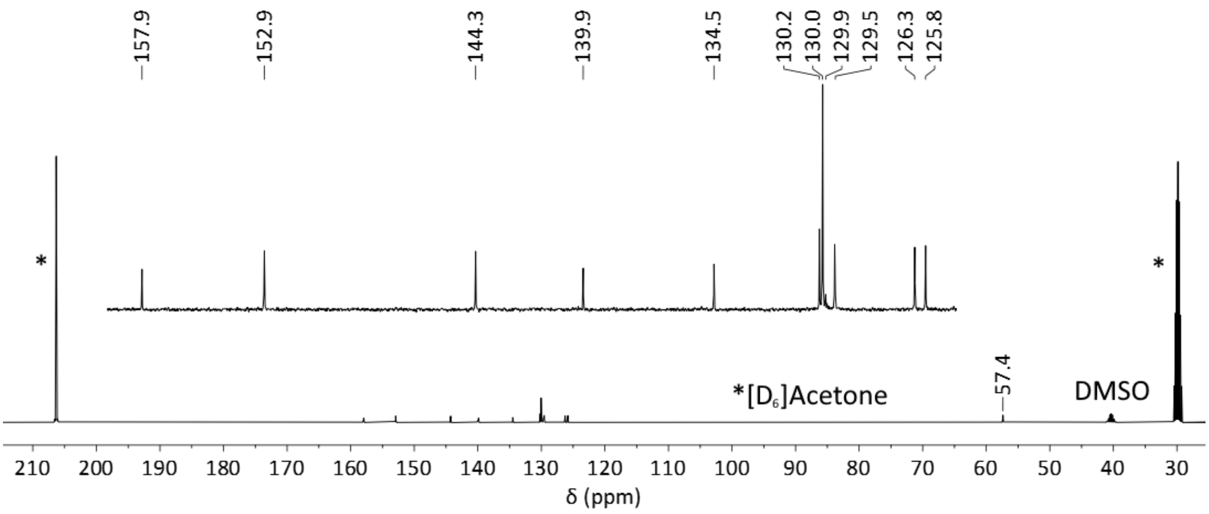

Figure 1.6 ^{13}C NMR spectrum spectra (100 MHz, [D$_6$]Acetone, 298 K) of $\mathbf{2_{AA}2_{DD}}$.

CCDC#: 2157804. Vapour diffusion of diethyl ether into a solution of $\mathbf{2_{AA}2_{DD}}$ and 9-methylanthracene in acetonitrile gave yellow crystals of ([9-methylanthracene⊂Pd($\mathbf{2_{AA}}$)($\mathbf{2_{DD}}$)](BF_4)$_2$). X-ray data were collected at 150 K on an Agilent Technologies Supernova system using Cu Kα radiation with exposures over 1.0°, and data were treated using CrysAlisPro[6] software. The structure was solved using SHELXT within OLEX2 and weighted full-matrix refinement on F^2 was carried out using SHELXL-97[7] running within the OLEX2[8] package. All non-hydrogen atoms were refined anisotropically. Hydrogen atoms attached to carbons were placed in calculated positions and refined using a riding model. The structure was solved in the monoclinic space group $P2_1/c$ and refined to an R_1 value of 7.1%. The asymmetric unit contained two complexes and four tetrafluoroborate counterions and four 9-methylanthracene molecules. Disorder within the lattice was modelled using the DFIX, DANG, FLAT, RIGU, SIMU, ISOR and PART commands. The PART command resolved disorder in one BF_4^- counterion, and one 9-methylanthracene molecule.

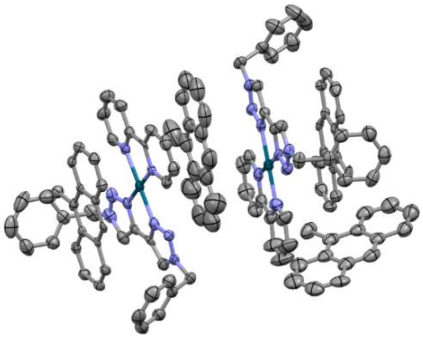

Figure 1.7 Mercury ellipsoid plot of the asymmetric unit of $\mathbf{2_{AA}2_{DD}}$. Ellipsoids shown at 50% probability level. Colour scheme: carbon grey, nitrogen blue, palladium dark blue. Hydrogen atoms and BF_4 counterions omitted for clarity.

Empirical formula	$C_{50.5}H_{42}B_2F_8N_8Pd$
Formula weight	1040.94
Temperature/K	150.01(10)
Crystal system	monoclinic
Space group	$P2_1/c$
a/Å	19.0390(3)
b/Å	24.3013(5)
c/Å	20.6293(4)
α/°	90
β/°	95.083(2)
γ/°	90
Volume/Å^3	9507.1(3)
Z	8
ρ_{calc}g/cm^3	1.455
μ/mm^{-1}	3.805
F(000)	4232.0
Crystal size/mm^3	0.3 × 0.05 × 0.05
Radiation	Cu Kα (λ = 1.54184)
2Θ range for data collection/°	7.064 to 142.848
Index ranges	-23 ≤ h ≤ 23, -29 ≤ k ≤ 28, -24 ≤ l ≤ 25
Reflections collected	53617
Independent reflections	18111 [R_{int} = 0.0583, R_{sigma} = 0.0898]
Data/restraints/parameters	18111/2095/1409
Goodness-of-fit on F^2	0.985
Final R indexes [I>=2σ (I)]	R_1 = 0.0714, wR_2 = 0.1681
Final R indexes [all data]	R_1 = 0.1150, wR_2 = 0.2013
Largest diff. peak/hole / e Å^{-3}	1.39/-1.20

1.3.4. $2_{DA}2_{AD}$

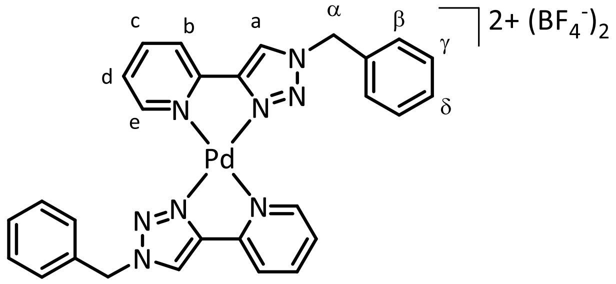

Characterisation was consistent with previous data.[4]

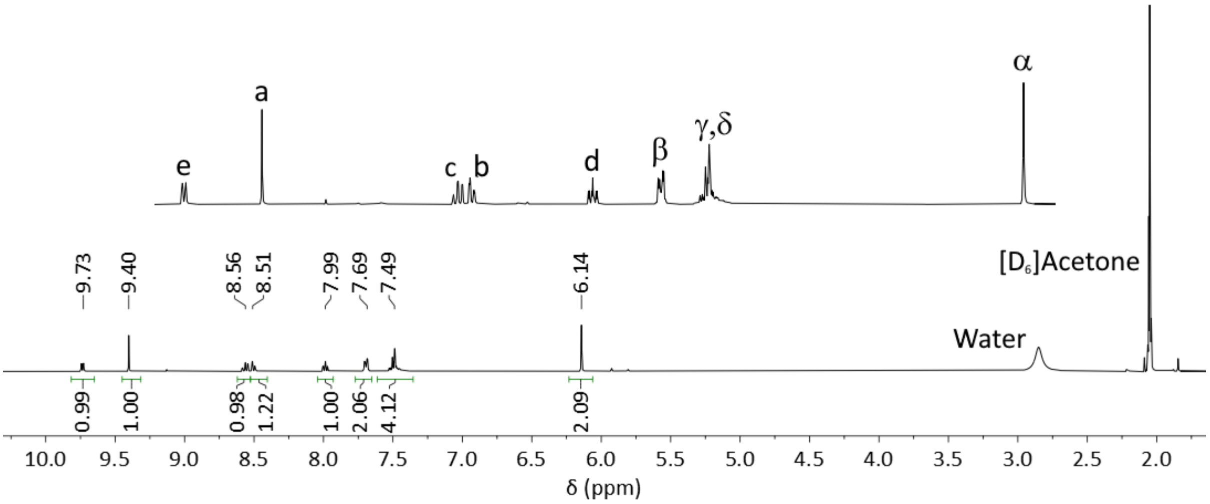

Figure 1.8 ^{1}H NMR spectrum (400 MHz, [D$_6$]Acetone, 298 K) of $\mathbf{2_{DA}2_{AD}}$.

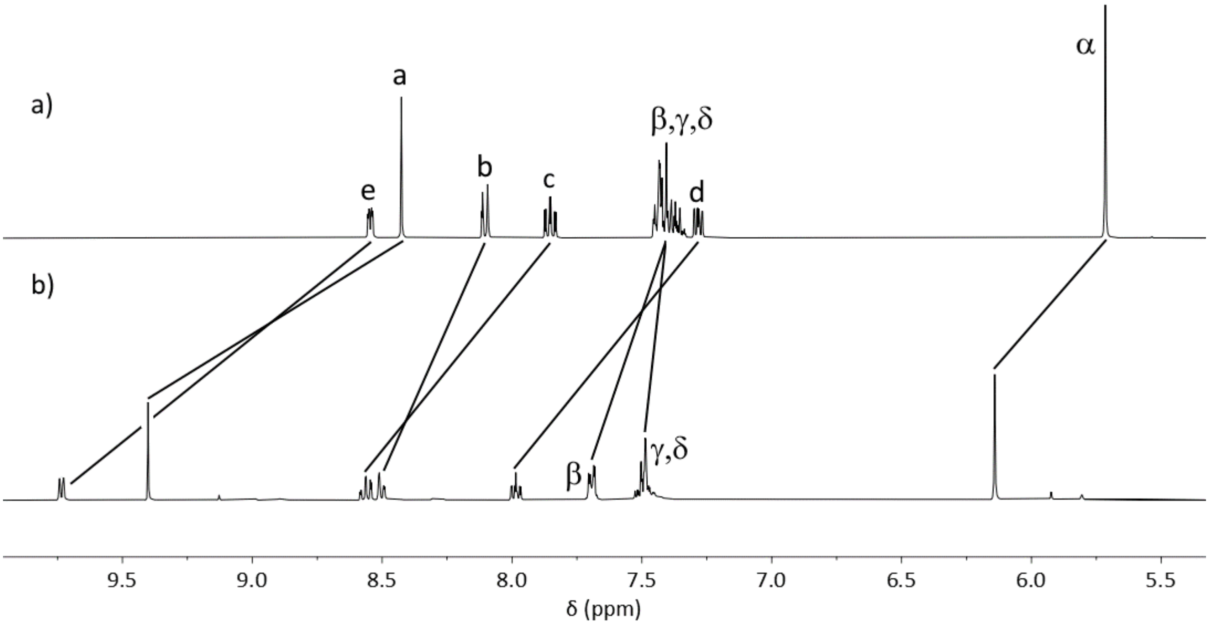

Figure 1.9 Stacked partial ^{1}H NMR spectra (400 MHz, [D$_6$]Acetone, 298 K) of a) $\mathbf{2_{DA}}$, and b) $\mathbf{2_{DA}2_{AD}}$.

1.3.5. $\mathbf{2_{Am}2_{Am}}$

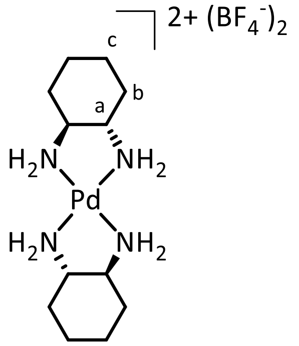

$[Pd(CH_3CN)_4](BF_4)_2$: 10.0 mg, 22.5 µmol. $\mathbf{2_{Am}}$: 5.14 mg, 45.0 µmol. Yield: 8.12 mg, 15.9 µmol, 71%. ^{1}H NMR (400 MHz, [D$_6$]DMSO, 298 K) δ: 5.10 (1H, d, J = 7.9 Hz, NH_{ax}), 4.46 (1H, t, J = 10.9 Hz, NH_{eq}), 2.26 (1H, br, H_a), 1.86 (1H, d, J = 10.5 Hz, H_{bax}), 1.55 (1H, d, J = 9.0 Hz, H_{cax}), 1.30 – 1.27 (1H, m, H_{beq}), 1.02 (1H, t, J = 9.6 Hz, H_{ceq}). ^{13}C NMR (100 MHz, [D$_6$]DMSO, 298 K) δ: 57.9, 30.8, 22.0. HR ESI-MS (DMSO/Acetonitrile) m/z = 333.1277 [M - H]$^+$ (calc. for $Pd(C_6H_{14}N_2)(C_6H_{13}N_2)$, 333.1276).

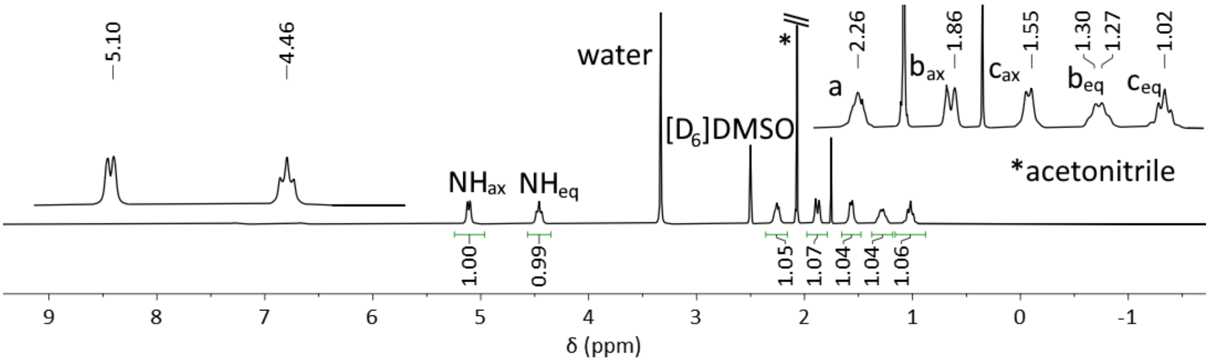

Figure 1.10 ^{1}H NMR spectrum (400 MHz, [D$_6$]DMSO, 298 K) of $\mathbf{2_{Am}2_{Am}}$.

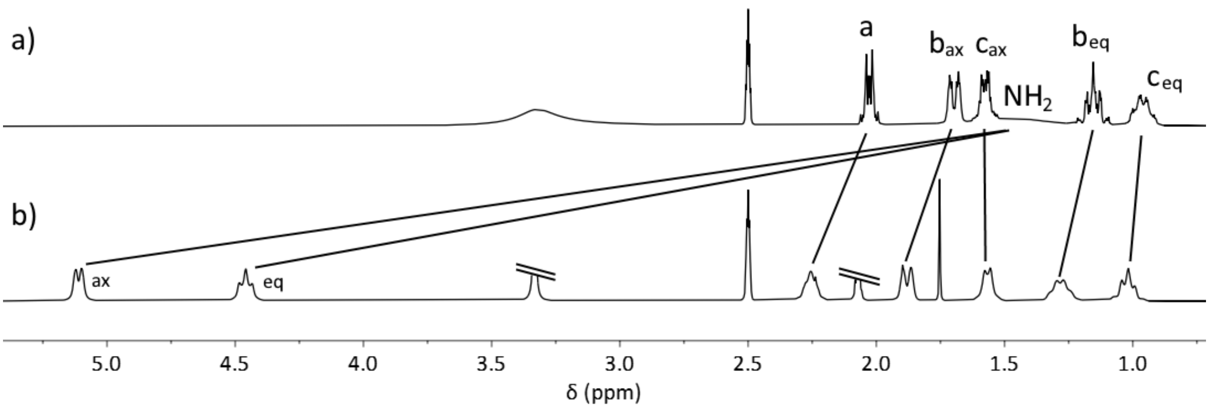

Figure 1.11 Stacked partial ^{1}H NMR spectra (400 MHz, [D$_6$]DMSO, 298 K) of a) $\mathbf{2_{Am}}$, and b) $\mathbf{2_{Am}2_{Am}}$.

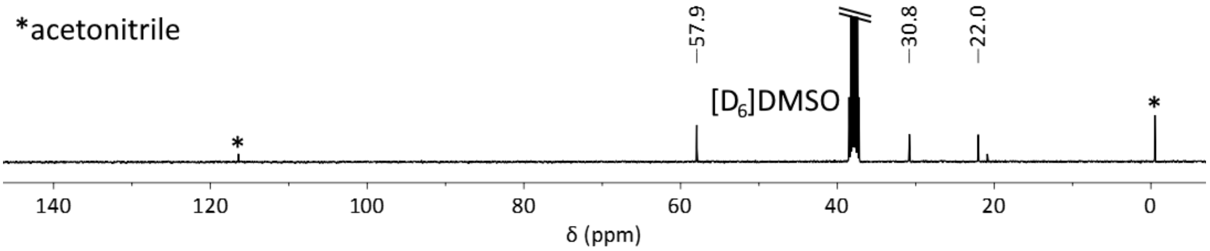

Figure 1.12 ^{13}C NMR spectrum spectra (100 MHz, [D$_6$]DMSO, 298 K) of $\mathbf{2_{Am}2_{Am}}$.

1.3.6. $3_{AA}1_{DD}$

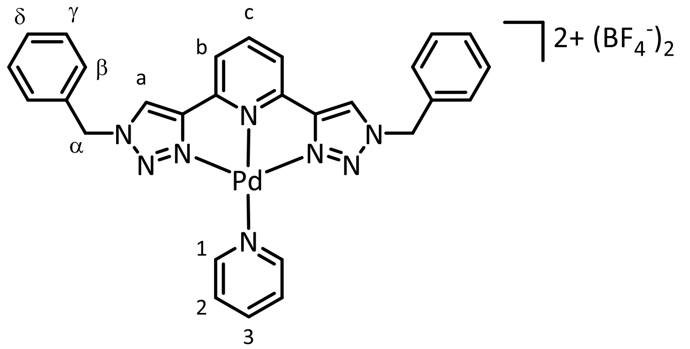

$[Pd(CH_3CN)_4](BF_4)_2$: 10.0 mg, 22.5 µmol. $\mathbf{3_{AA}}$[5]: 8.88 mg, 22.5 µmol. $\mathbf{1_{DD}}$: 1.79 mg, 22.5 µmol. Yield: 12.70 mg, 16.88 µmol, 75%. ^{1}H NMR (400 MHz, [D$_6$]Acetone, 298 K) δ: 9.28 (2H, s, H$_a$), 9.18 (2H, d, J = 6.48 Hz, H$_1$), 8.57 (1H, t, J = 7.7 Hz, H$_c$), 8.43 (1H, t, J = 7.83 Hz, H$_3$), 8.33 (2H, d, J = 7.95 Hz, H$_b$), 7.97 (2H, t, J = 6.48 Hz, H$_2$), 7.56 (4H, m, H$_\beta$), 7.47 (6H, m, H$_{\gamma,\delta}$), 5.95 (4H, s, H$_\alpha$). ^{13}C NMR (100 MHz, [D$_6$]Acetone, 298 K) δ: 153.6, 150.9, 148.9, 146.0, 142.8, 134.3, 130.3, 130.2, 130.0, 128.4, 128.1, 123.0, 57.5. HR ESI-MS (DMSO/Acetonitrile) m/z = 518.0744 [M + F]$^+$ (calc. for $Pd(C_{23}H_{19}N_7)F$, 518.729).

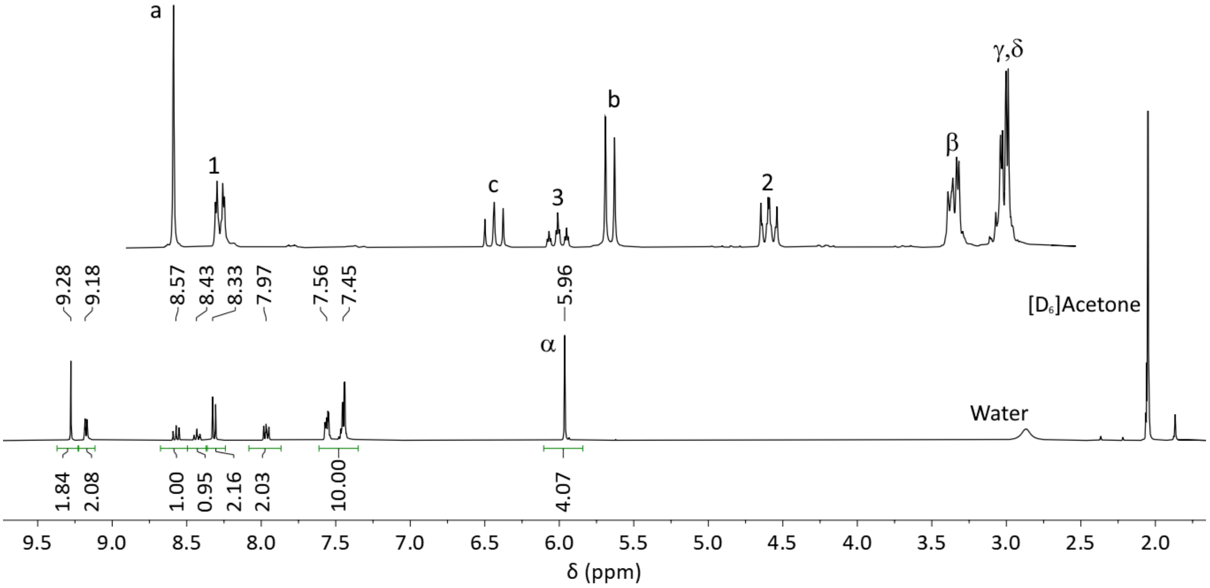

Figure 1.13 ^{1}H NMR spectrum (400 MHz, [D6]Acetone, 298 K) of $\mathbf{3_{AA}1_{DD}}$.

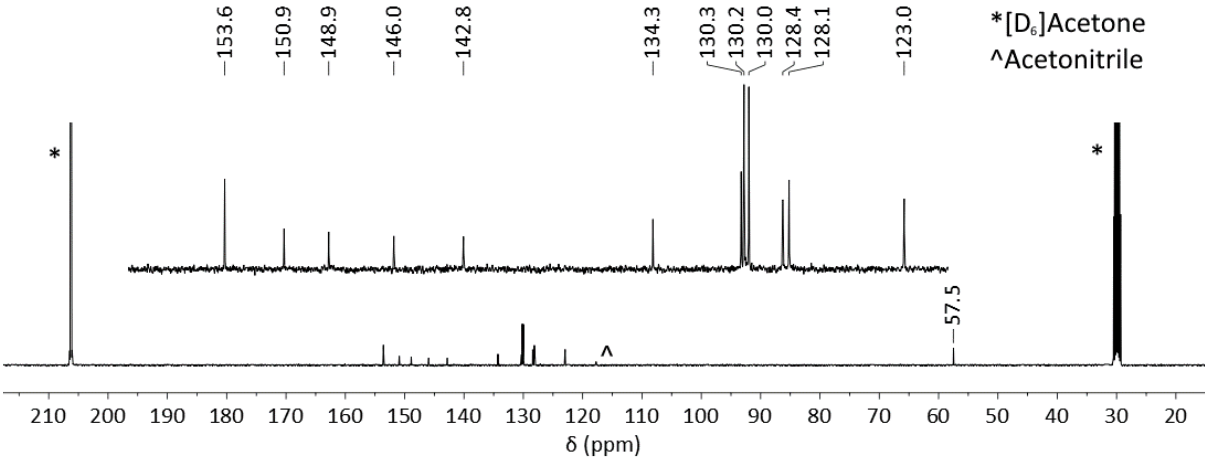

Figure 1.14 ^{13}C NMR spectrum spectra (100 MHz, [D$_6$]Acetone, 298 K) of $\mathbf{3_{AA}1_{DD}}$.

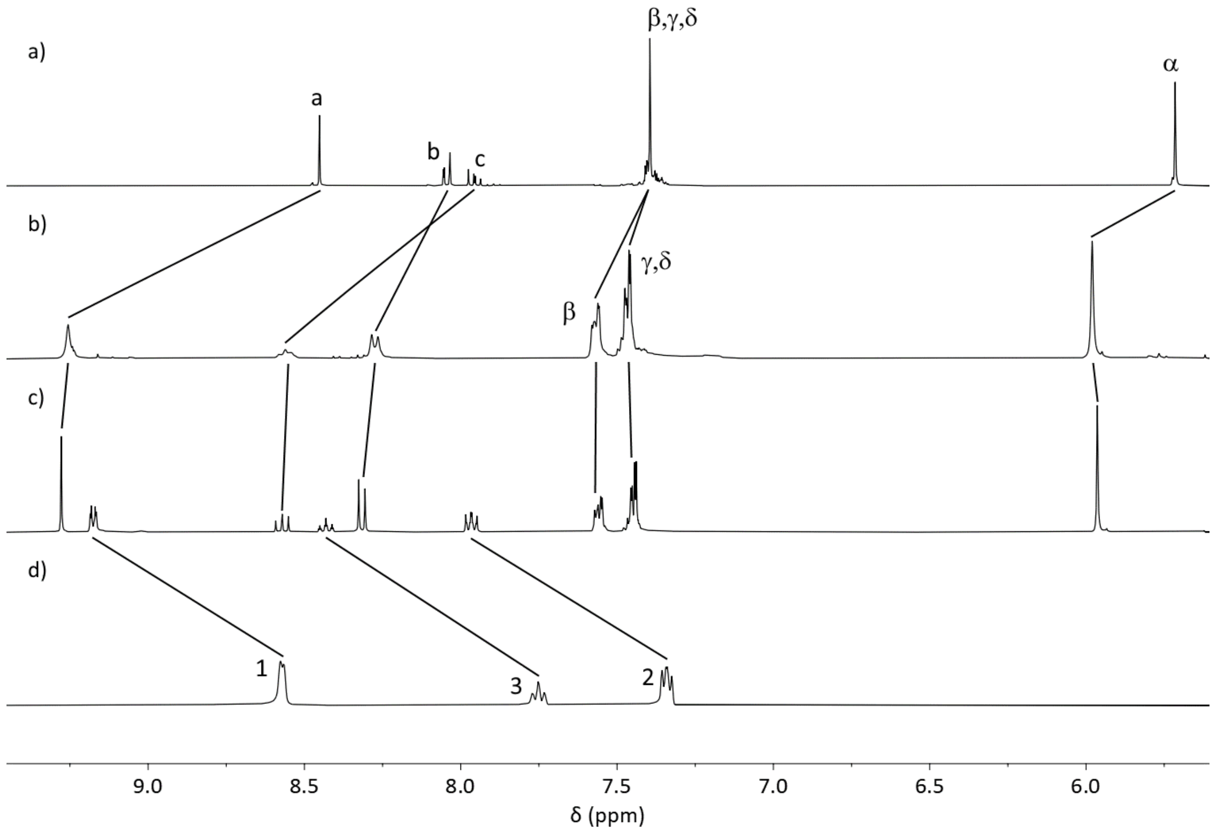

Figure 1.15 Stacked partial ^{1}H NMR spectra (400 MHz, [D$_6$]Acetone, 298 K) of a) $\mathbf{3_{AA}}$, b) [Pd($\mathbf{3_{AA}}$)([D$_6$]Acetone)]$^{2+}$, c) $\mathbf{3_{AA}1_{DD}}$, and d) $\mathbf{1_{DD}}$.

CCDC#: 2157805. Vapour diffusion of diethyl ether into a solution of $3_{AA}1_{DD}$ and anthracene in acetonitrile gave yellow crystals of ([anthracene⊂Pd($\mathbf{3_{AA}}$)($\mathbf{1_{DD}}$)](BF_4)$_2$). X-ray data were collected at 150 K on an Agilent Technologies Supernova system using Cu Kα radiation with exposures over 1.0°, and data were treated using CrysAlisPro[6] software. The structure was solved using SHELXT within OLEX2 and weighted full-matrix refinement on F^2 was carried out using SHELXL-97[7] running within the OLEX2[8] package. All non-hydrogen atoms were refined anisotropically. Hydrogen atoms attached to carbons were placed in calculated positions and refined using a riding model. The structure was solved in the monoclinic space group $P2_1/c$ and refined to an R_1 value of 3.4%. The asymmetric unit contained one complex and two tetrafluoroborate counterions and two half anthracene molecules.

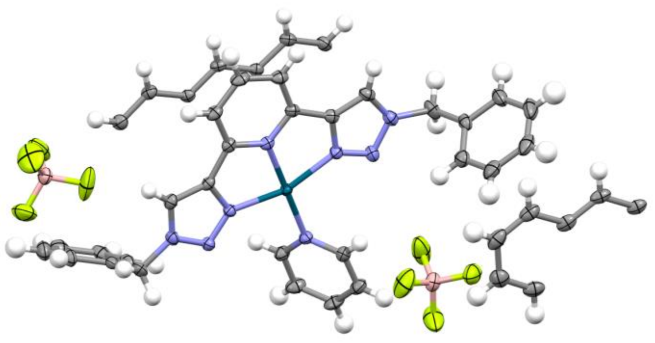

Figure 1.16 Mercury ellipsoid plot of the asymmetric unit of $\mathbf{3_{AA}1_{DD}}$. Ellipsoids shown at 50% probability level. Colour scheme: carbon grey, hydrogen white, boron salmon, fluorine lime, nitrogen blue, palladium dark blue.

Empirical formula	$C_{42}H_{34}B_2F_8N_8Pd$
Formula weight	930.843
Temperature/K	150.00(10)
Crystal system	monoclinic
Space group	$P2_1/c$
a/Å	13.5152(1)
b/Å	15.4672(1)
c/Å	18.9215(1)
α/°	90
β/°	101.241(1)
γ/°	90
Volume/Å^3	3879.51(5)
Z	4
ρ_{calc}g/cm^3	1.594
μ/mm^{-1}	4.583
F(000)	1887.8
Crystal size/mm^3	0.288 × 0.174 × 0.147
Radiation	Cu Kα (λ = 1.54184)
2Θ range for data collection/°	7.44 to 147.58
Index ranges	-16 ≤ h ≤ 16, -18 ≤ k ≤ 19, -23 ≤ l ≤ 16
Reflections collected	23022
Independent reflections	7697 [R_{int} = 0.0201, R_{sigma} = 0.0184]
Data/restraints/parameters	7697/0/550
Goodness-of-fit on F^2	1.012
Final R indexes [I>=2σ (I)]	R_1 = 0.0335, wR_2 = 0.1088
Final R indexes [all data]	R_1 = 0.0346, wR_2 = 0.1107
Largest diff. peak/hole / e Å^{-3}	0.73/-0.95

1.3.7. $3_{AD}1_{DA}$

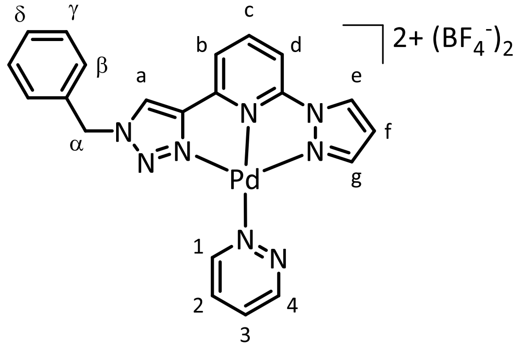

$[Pd(CH_3CN)_4](BF_4)_2$: 10.0 mg, 22.5 µmol. **3**$_{AD}$: 6.80 mg, 22.5 µmol. **1**$_{DA}$: 1.80 mg, 22.5 µmol. Yield: 12.06 mg, 18.22 µmol, 81%. ^{1}H NMR (400 MHz, [D$_6$]Acetone, 298 K) δ: 10.03 (1H, m, H$_1$), 9.67 (1H, m, H$_4$), 9.38 (1H, s, H$_a$), 9.25 (1H, d, J = 3.18 Hz, H$_e$), 8.74 (1H, t, J = 7.83 Hz, H$_c$), 8.62 (1H, d, J = 2.44 Hz, H$_g$), 8.47 (2H, m, H$_{2,3}$), 8.37 (4H, m, H$_{b,d}$), 7.60 (1H, m, H$_\beta$), 7.47 (3H, m, H$_{\gamma,\delta}$), 7.13 (1H, t, J = 3.18 Hz, H$_f$), 6.05 (2H, s, H$_\alpha$). ^{13}C NMR (100 MHz, [D$_6$]DMSO, 298 K) δ: 171.5, 156.6, 154.6, 148.9, 147.9, 146.3, 146.1, 145.1, 134.5, 133.5, 132.7, 129.2, 129.2, 128.8, 127.8, 119.8, 112.1, 110.3, 55.9. HR ESI-MS (DMSO/Acetonitrile) m/z = 427.0329 [M + F]$^+$ (calc. for $Pd(C_{17}H_{14}N_6)F$, 427.0305).

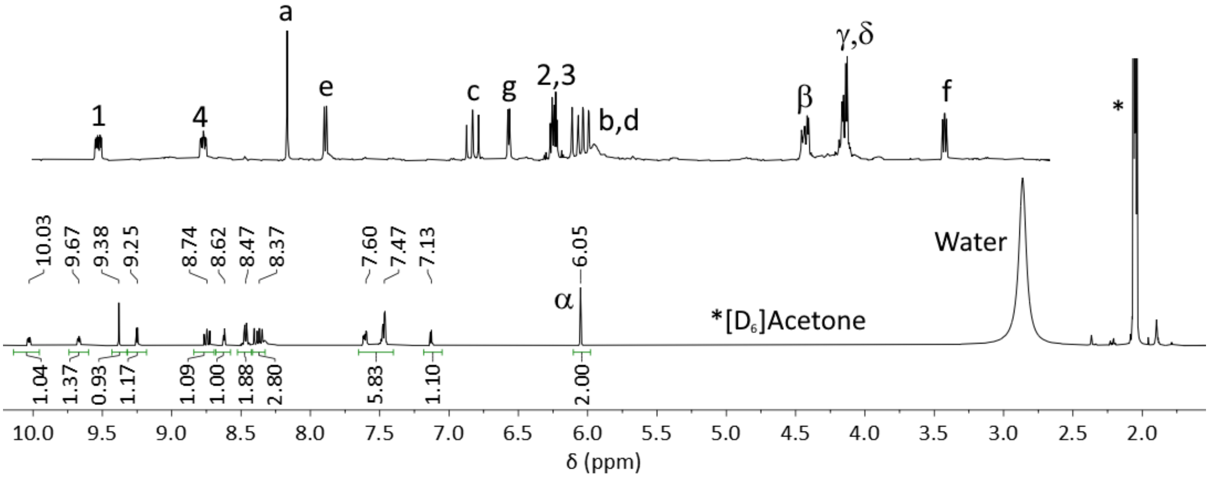

Figure 1.17 ^{1}H NMR spectrum (400 MHz, [D$_6$]Acetone, 298 K) of **3**$_{AD}$**1**$_{DA}$.

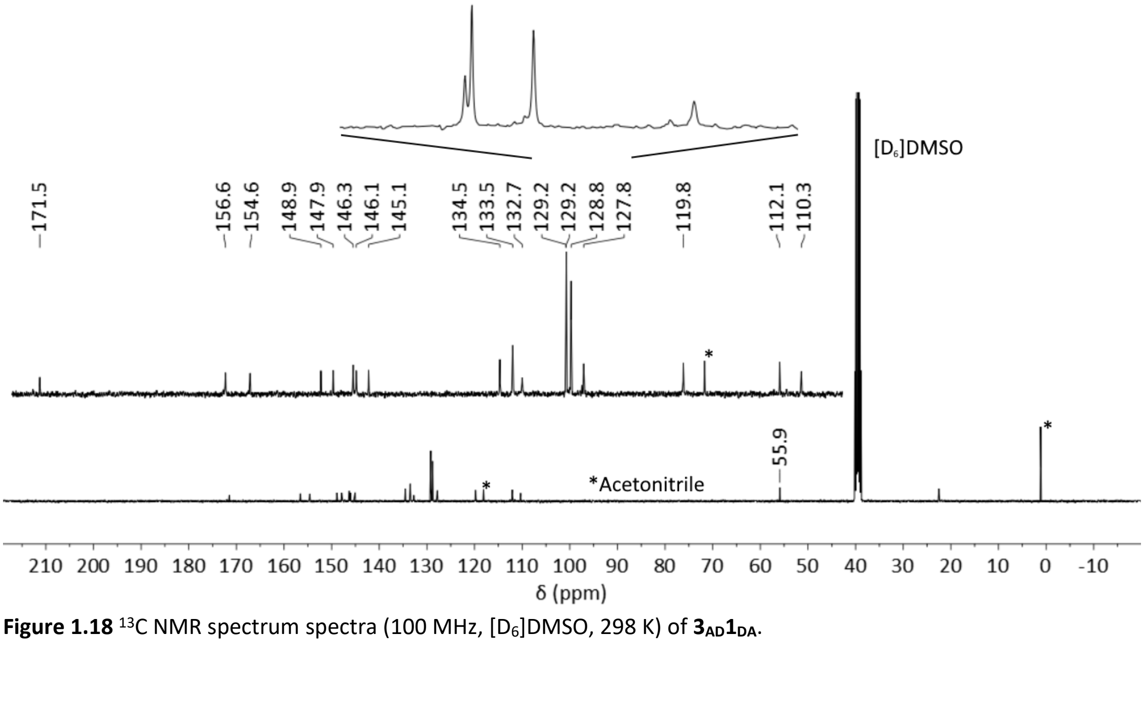

Figure 1.18 ^{13}C NMR spectrum spectra (100 MHz, [D$_6$]DMSO, 298 K) of **3**$_{AD}$**1**$_{DA}$.

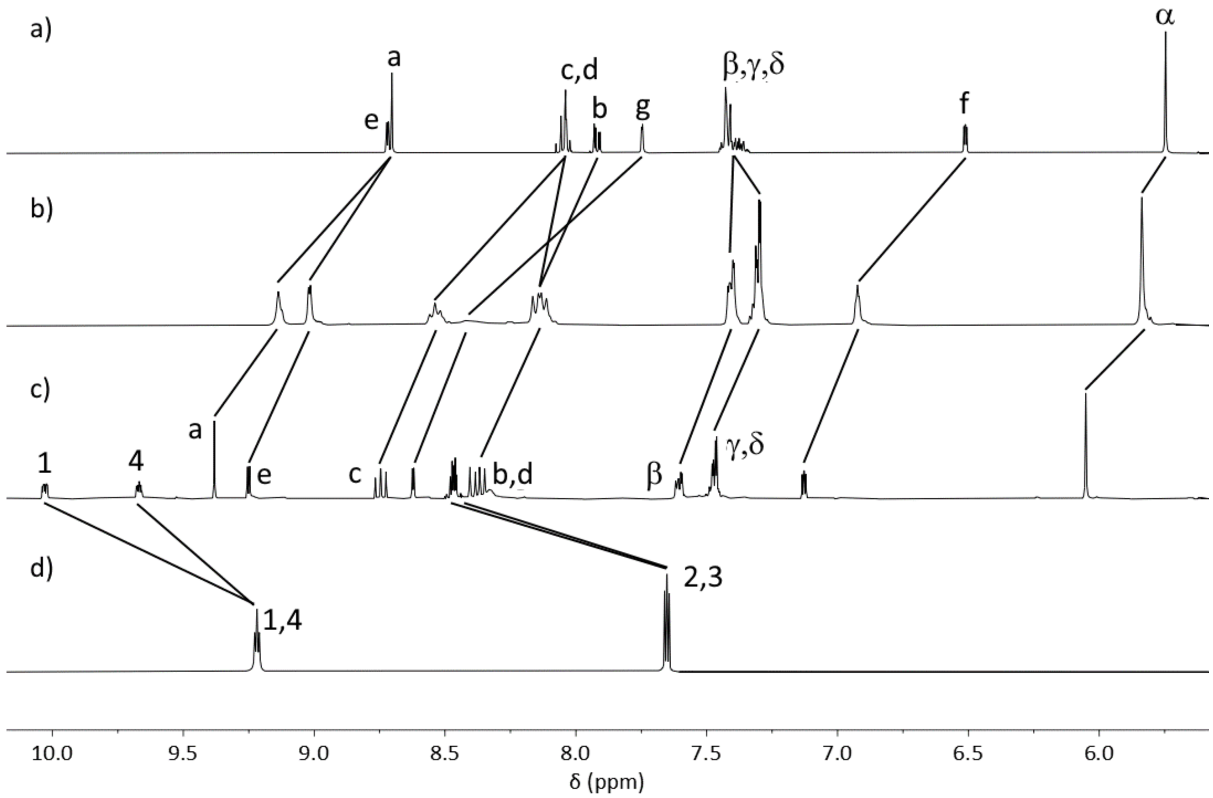

Figure 1.19 Stacked partial ^{1}H NMR spectra (400 MHz, [D_6]Acetone, 298 K) of a) $\mathbf{3_{AD}}$, b) [Pd($\mathbf{3_{AD}}$)([D_6]Acetone)]$^{2+}$, c) $\mathbf{3_{AD}1_{DA}}$, and d) $\mathbf{1_{DA}}$.

CCDC#: 2157806. Vapour diffusion of diethyl ether into a solution of $3_{DA}1_{DA}$ in acetonitrile gave colourless crystals of ([Pd($\mathbf{3_{DA}}$)($\mathbf{1_{AD}}$)](BF_4)$_2$). X-ray data were collected at 150 K on an Agilent Technologies Supernova system using Cu Kα radiation with exposures over 1.0°, and data were treated using CrysAlisPro[6] software. The structure was solved using SHELXT within OLEX2 and weighted full-matrix refinement on F^2 was carried out using SHELXL-97[7] running within the OLEX2[8] package. All non-hydrogen atoms were refined anisotropically. Hydrogen atoms attached to carbons were placed in calculated positions and refined using a riding model. The structure was solved in the monoclinic space group $P2_1$/n and refined to an R_1 value of 2.5%. The asymmetric unit contained one complex and two tetrafluoroborate counterions.

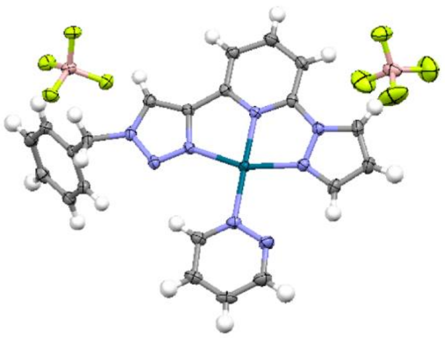

Figure 1.20 Mercury ellipsoid plot of the asymmetric unit of $\mathbf{3_{AD}1_{DA}}$. Ellipsoids shown at 50% probability level. Colour scheme: carbon grey, hydrogen white, boron salmon, fluorine lime, nitrogen blue, palladium dark blue.

Empirical formula	$C_{21}H_{18}B_2F_8N_8Pd$
Formula weight	662.45
Temperature/K	150.01(10)
Crystal system	monoclinic
Space group	$P2_1/n$
a/Å	9.63320(10)
b/Å	8.11500(10)
c/Å	30.9091(3)
α/°	90
β/°	98.5340(10)
γ/°	90
Volume/Å^3	2389.52(5)
Z	4
ρ_{calc}g/cm^3	1.841
μ/mm^{-1}	7.123
F(000)	1312.0
Crystal size/mm^3	0.196 × 0.147 × 0.055
Radiation	Cu Kα (λ = 1.54184)
2Θ range for data collection/°	9.304 to 142.094
Index ranges	-11 ≤ h ≤ 11, -6 ≤ k ≤ 9, -37 ≤ l ≤ 36
Reflections collected	18122
Independent reflections	4555 [R_{int} = 0.0143, R_{sigma} = 0.0101]
Data/restraints/parameters	4555/0/361
Goodness-of-fit on F^2	1.009
Final R indexes [I>=2σ (I)]	R_1 = 0.0251, wR_2 = 0.0682
Final R indexes [all data]	R_1 = 0.0253, wR_2 = 0.0684
Largest diff. peak/hole / e Å^{-3}	1.21/-0.73

1.4. $\mathbf{3_{AA}1_{DA}}$

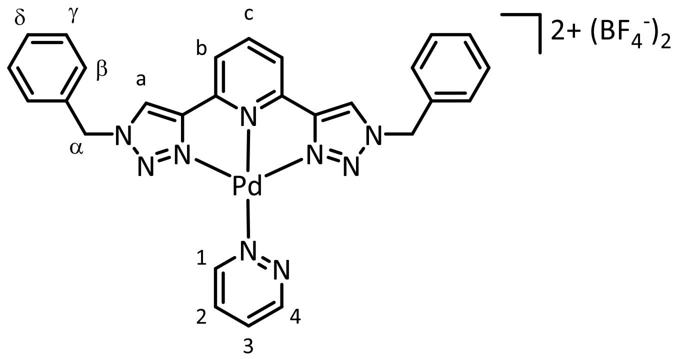

$[Pd(CH_3CN)_4](BF_4)_2$: 10.0 mg, 22.5 µmol. $\mathbf{3_{AA}}$[5]: 8.88 mg, 22.5 µmol. $\mathbf{1_{DA}}$: 1.80 mg, 22.5 µmol. Yield: 13.37 mg, 17.78 µmol, 75%. ^{1}H NMR (400 MHz, [D$_6$]DMSO, 298 K) δ: 9.90 (1H, br, H$_1$), 9.65 (1H, br, H$_4$), 9.50 (2H, s, H$_a$), 8.68 (1H, t, J = 7.9 Hz, H$_c$), 8.50 – 8.40 (4H, m, H$_{2,3,b}$), 7.64 – 7.49 (10H, m, H$_{\beta,\gamma,\delta}$), 6.02 (4H, s, H$_\alpha$). ^{13}C NMR (100 MHz, [D$_6$]DMSO, 298 K) δ: 169.8, 155.4, 152.7, 147.6, 145.6, 143.3, 131.9, 131.4, 131.1, 127.5, 127.1, 126.0, 120.2, 54.1. HR ESI-MS (DMSO/Acetonitrile) m/z = 488.0574 [M - bz]$^+$ (calc. for $Pd(C_{16}H_{12}N_7)(C_4H_4N_2)$, 488.0571); 666.1168 [M + BF$_4$]$^+$ (calc. for $Pd(C_{23}H_{19}N_7)(C_4H_4N_2)BF_4$, 666.1154).

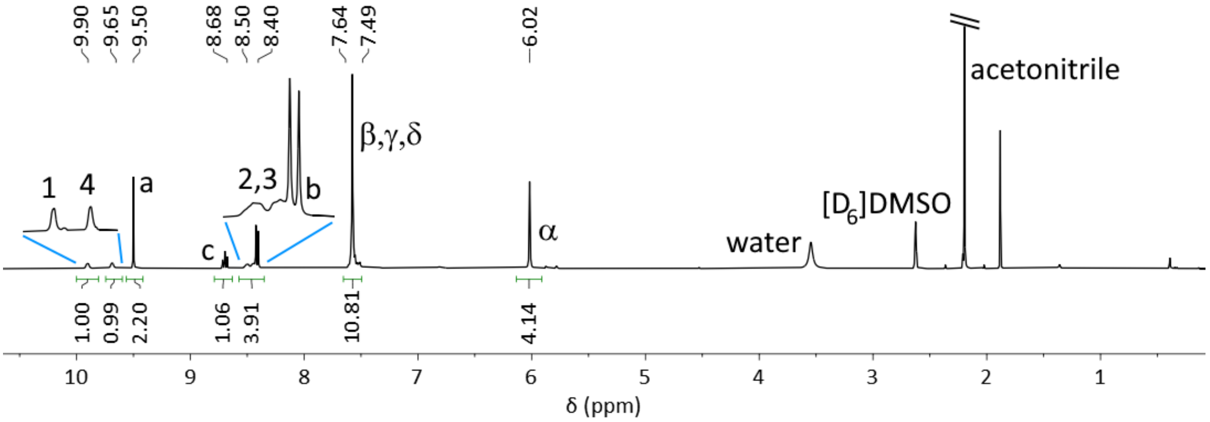

Figure 1.21 ^{1}H NMR spectrum (400 MHz, [D$_6$]DMSO, 298 K) of $\mathbf{3_{AA}1_{DA}}$.

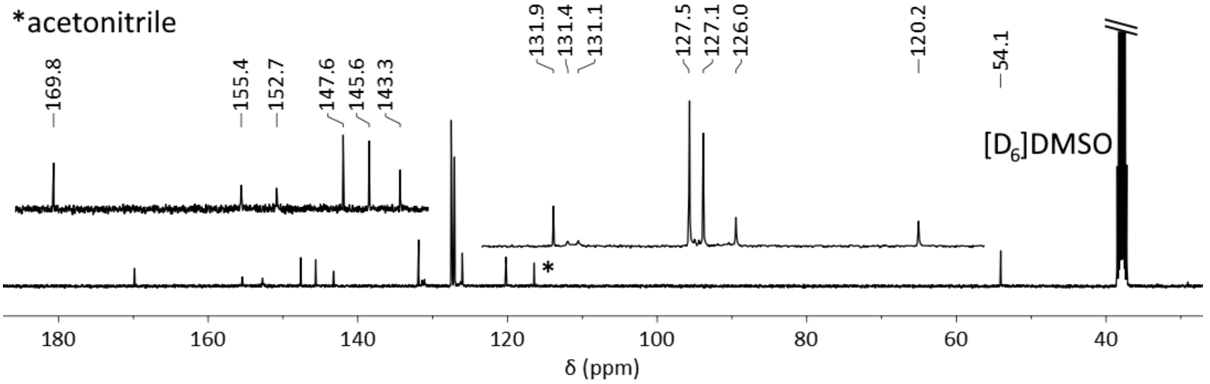

Figure 1.22 ^{13}C NMR spectrum (100 MHz, [D$_6$]DMSO, 298 K) of $\mathbf{3_{AA}1_{DA}}$.

1.5. $3_{AD}1_{DD}$

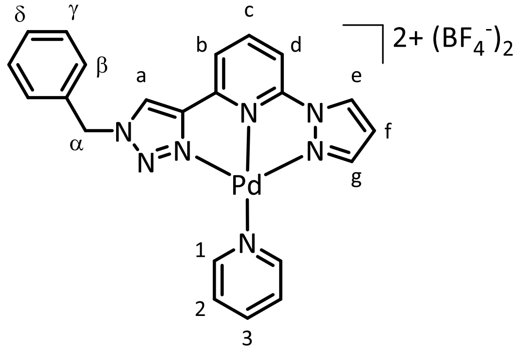

$[Pd(CH_3CN)_4](BF_4)_2$: 10.0 mg, 22.5 µmol. $\mathbf{3_{AD}}$: 6.80 mg, 22.5 µmol. $\mathbf{1_{DD}}$: 1.79 mg, 22.5 µmol. Yield: 12.38 mg, 18.67 µmol, 83%. ^{1}H NMR (400 MHz, $[D_6]$DMSO, 298 K) *δ*: 9.24 (1H, s, H_a), 9.21 (1H, d, *J* = 3.4 Hz, H_e), 8.85 (2H, d, *J* = 6.6 Hz, H_1), 8.56 (1H, t, *J* = 8.1 Hz, H_c), 8.21 – 8.08 (4H, m, $H_{3,b,d,g}$), 7.75 (2H, t, *J* = 6.5 Hz, H_2), 7.31 – 7.27 (5H, m, $H_{\beta,\gamma,\delta}$), 6.95 (1H, t, *J* = 3.0 Hz, H_f), 5.74 (2H, s, H_α). ^{13}C NMR (100 MHz, $[D_6]$DMSO, 298 K) *δ*: 169.8, 150.6, 147.2, 146.1, 144.9, 144.7, 143.5, 140.0, 132.8, 131.8, 127.6, 127.1, 126.1, 125.6, 116.4, 110.5, 109.1, 54.1. HR ESI-MS (DMSO/Acetonitrile) m/z = 396.0199 [M - bz]$^+$ (calc. for $Pd(C_{10}H_7N_6)(C_4H_4N_2)$, 396.0195).

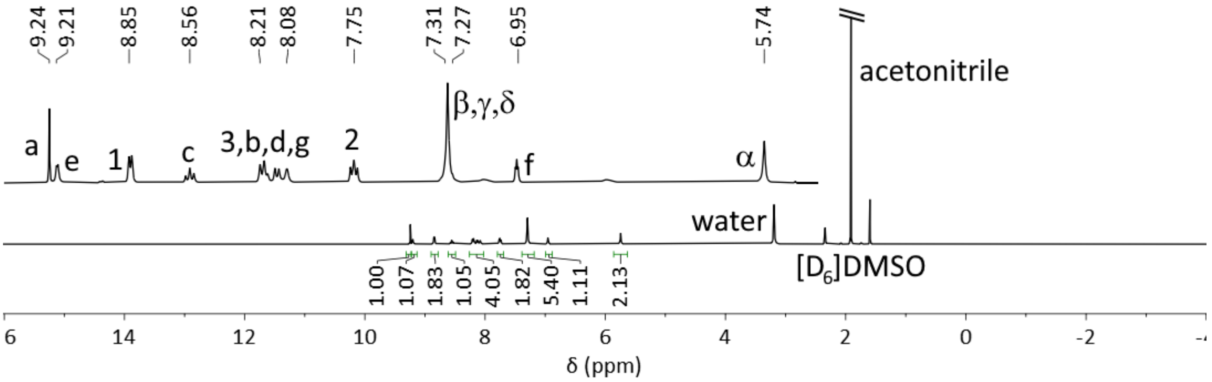

Figure 1.23 ^{1}H NMR spectrum (400 MHz, $[D_6]$DMSO, 298 K) of $\mathbf{3_{DA}1_{DD}}$.

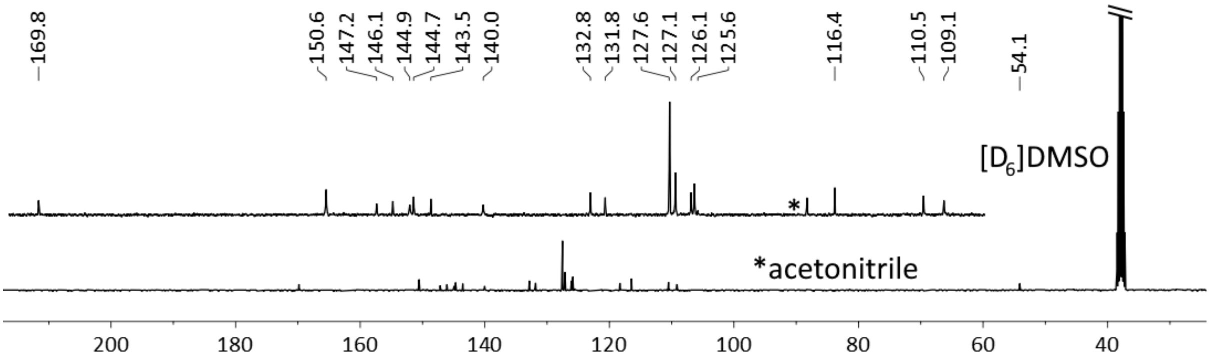

Figure 1.24 ^{13}C NMR spectrum (100 MHz, $[D_6]$DMSO, 298 K) of $\mathbf{3_{DA}1_{DD}}$.

2. Combinatorial studies

2.1. 1-to-1 combinations

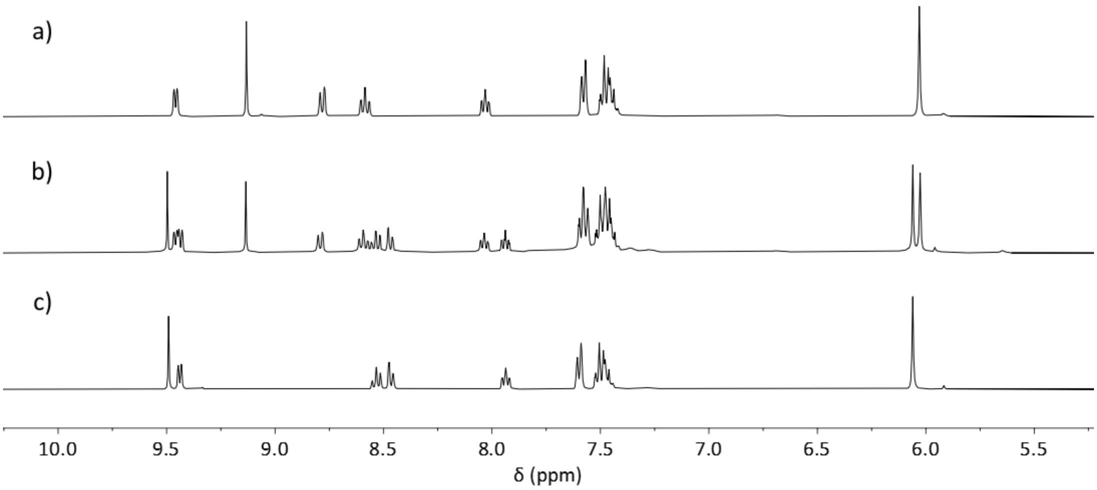

Figure 2.1 Stacked partial ^{1}H NMR spectra (400 MHz, $[D_6]$DMSO, 298 K) of a) $\mathbf{2_{AA}2_{DD}}$, b) $\mathbf{2_{AA}2_{DD}}$ and $\mathbf{2_{DA}2_{AD}}$, and c) $\mathbf{2_{DA}2_{AD}}$.

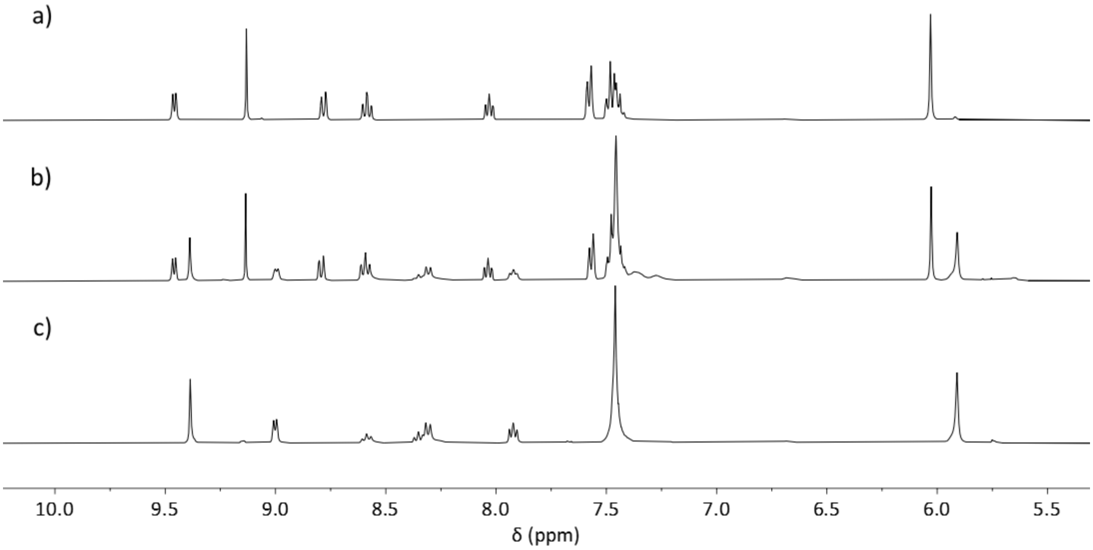

Figure 2.2 Stacked partial ^{1}H NMR spectra (400 MHz, $[D_6]$DMSO, 298 K) of a) $\mathbf{2_{AA}2_{DD}}$, b) $\mathbf{2_{AA}2_{DD}}$ and $\mathbf{3_{AA}1_{DD}}$, and c) $\mathbf{3_{AA}1_{DD}}$.

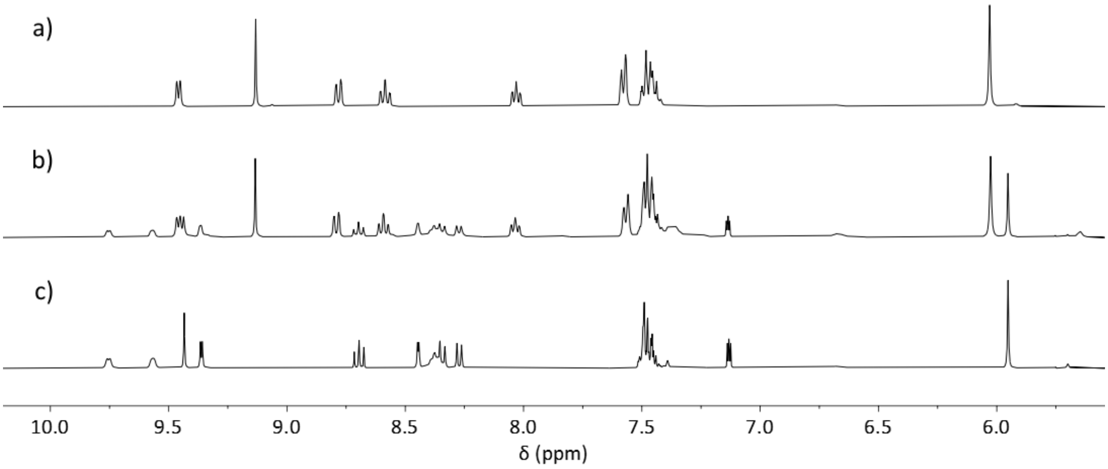

Figure 2.3 Stacked partial ^{1}H NMR spectra (400 MHz, $[D_6]$DMSO, 298 K) of a) $\mathbf{2_{AA}2_{DD}}$, b) $\mathbf{2_{AA}2_{DD}}$ and $\mathbf{3_{AD}1_{DA}}$, and c) $\mathbf{3_{AD}1_{DA}}$.

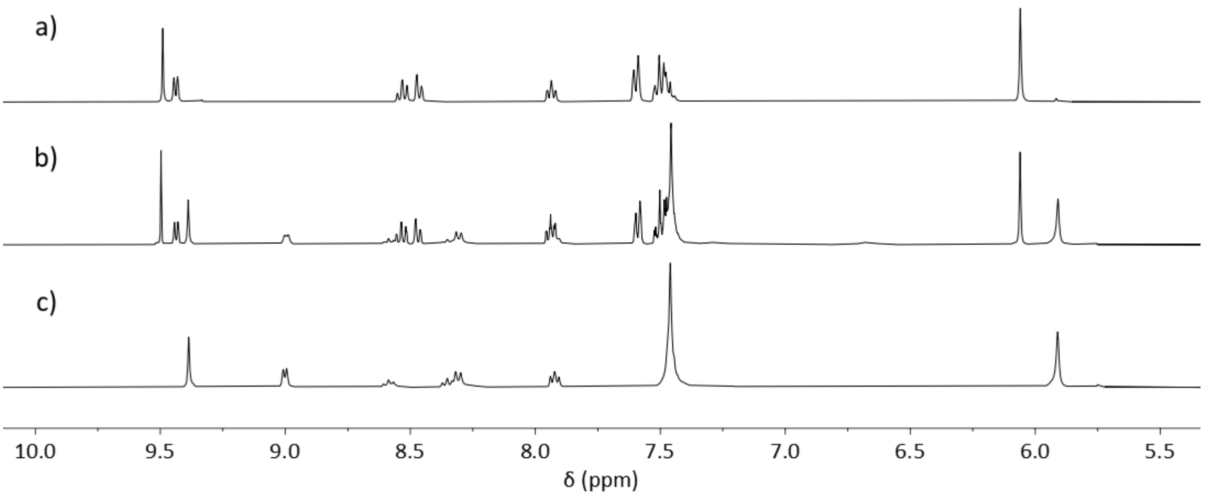

Figure 2.4 Stacked partial ^{1}H NMR spectra (400 MHz, [D$_6$]DMSO, 298 K) of a) $\mathbf{2_{AD}2_{DA}}$, b) $\mathbf{2_{AD}2_{DA}}$ and $\mathbf{3_{AA}1_{DD}}$, and c) $\mathbf{3_{AA}1_{DD}}$.

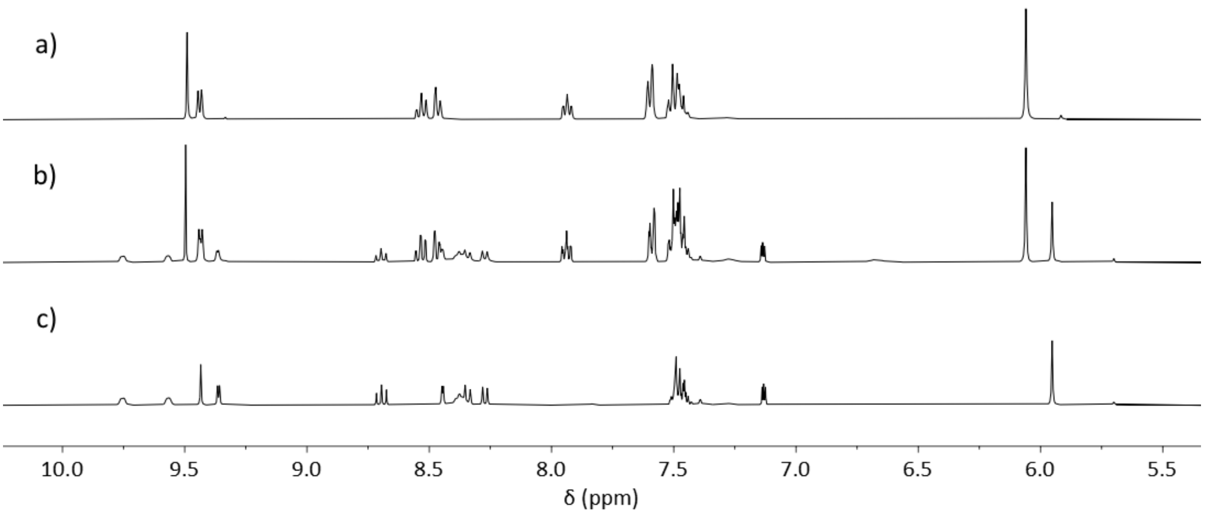

Figure 2.5 Stacked partial ^{1}H NMR spectra (400 MHz, [D$_6$]DMSO, 298 K) of a) $\mathbf{2_{AD}2_{DA}}$, b) $\mathbf{2_{AD}2_{DA}}$ and $\mathbf{3_{AD}1_{DA}}$, and c) $\mathbf{3_{AD}1_{DA}}$.

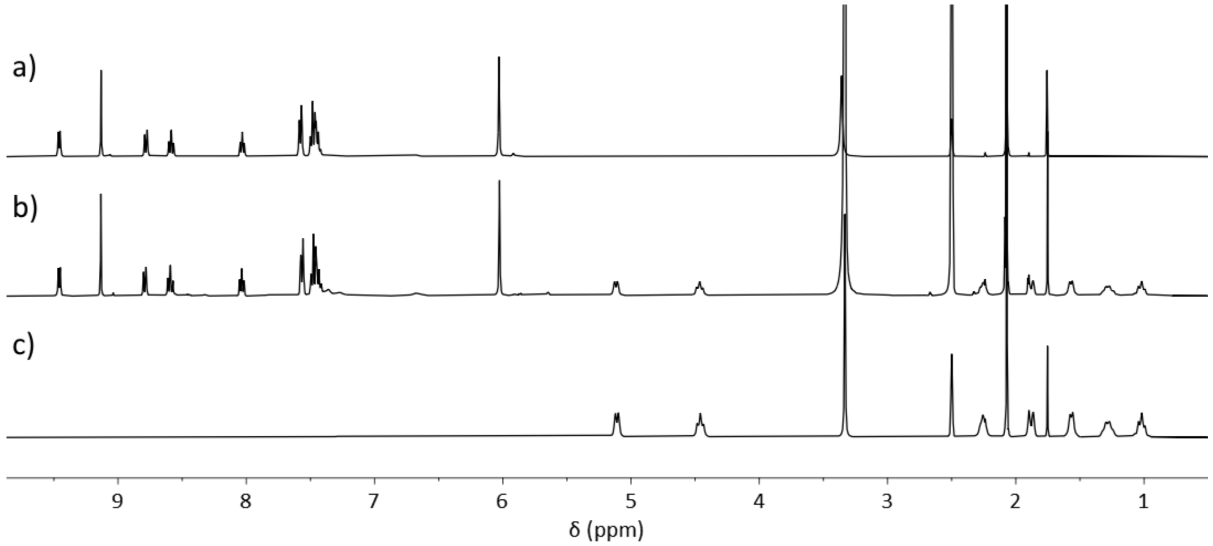

Figure 2.6 Stacked partial ^{1}H NMR spectra (400 MHz, [D$_6$]DMSO, 298 K) of a) $\mathbf{2_{AA}2_{DD}}$, b) $\mathbf{2_{AA}2_{DD}}$ and $\mathbf{2_{Am}2_{Am}}$, and c) $\mathbf{2_{Am}2_{Am}}$.

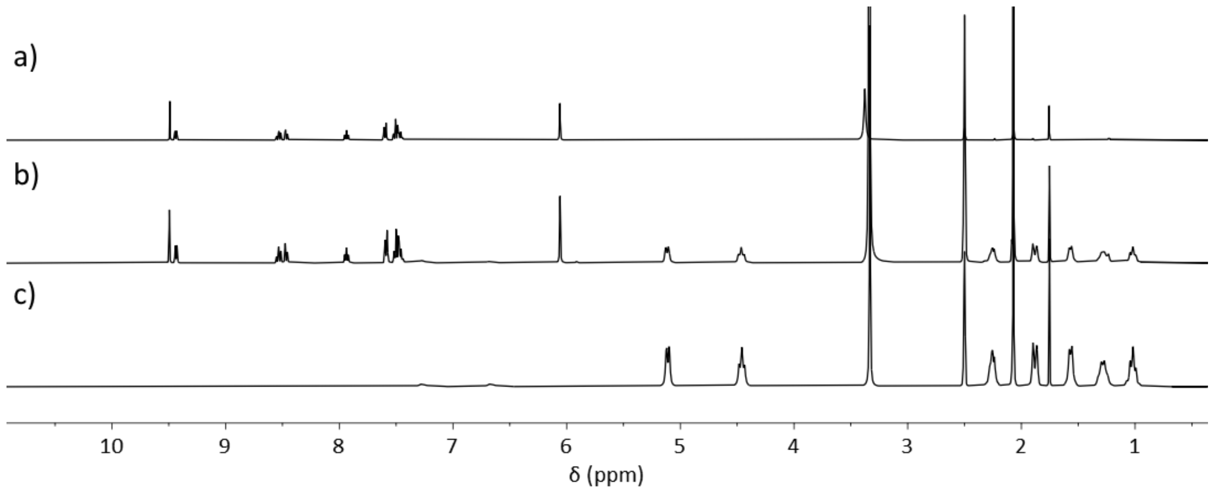

Figure 2.7 Stacked partial ^{1}H NMR spectra (400 MHz, [D$_6$]DMSO, 298 K) of a) $\mathbf{2_{DA}2_{AD}}$, b) $\mathbf{2_{DA}2_{AD}}$ and $\mathbf{2_{Am}2_{Am}}$, and c) $\mathbf{2_{Am}2_{Am}}$.

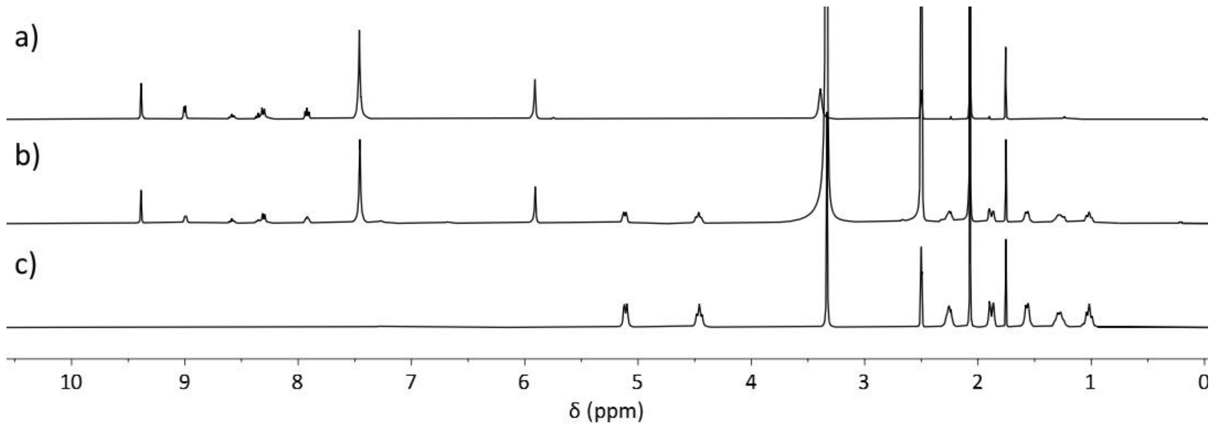

Figure 2.8 Stacked partial ^{1}H NMR spectra (400 MHz, [D$_6$]DMSO, 298 K) of a) $\mathbf{3_{AA}1_{DD}}$, b) $\mathbf{3_{AA}1_{DD}}$ and $\mathbf{2_{Am}2_{Am}}$, and c) $\mathbf{2_{Am}2_{Am}}$.

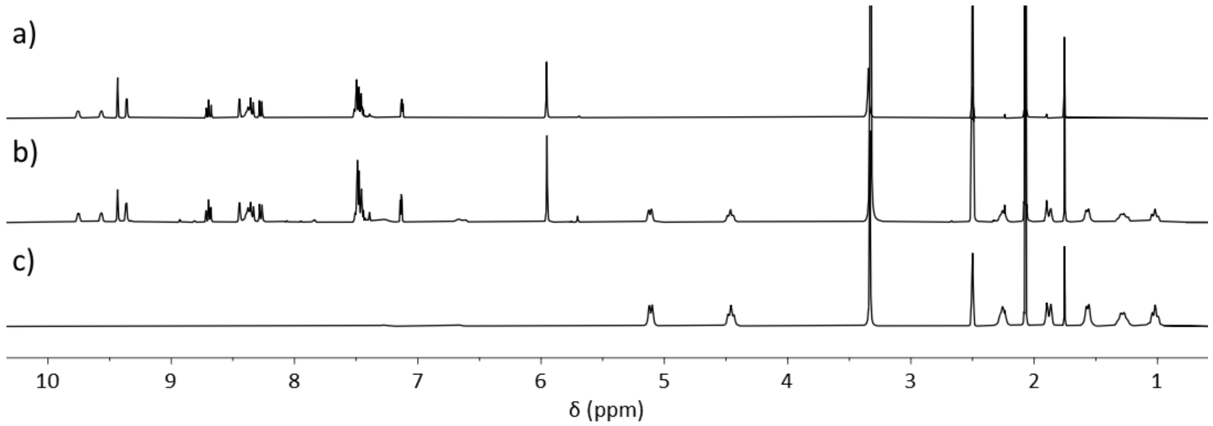

Figure 2.9 Stacked partial ^{1}H NMR spectra (400 MHz, [D$_6$]DMSO, 298 K) of a) $\mathbf{3_{DA}1_{AD}}$, b) $\mathbf{3_{DA}1_{AD}}$ and $\mathbf{2_{Am}2_{Am}}$, and c) $\mathbf{2_{Am}2_{Am}}$.

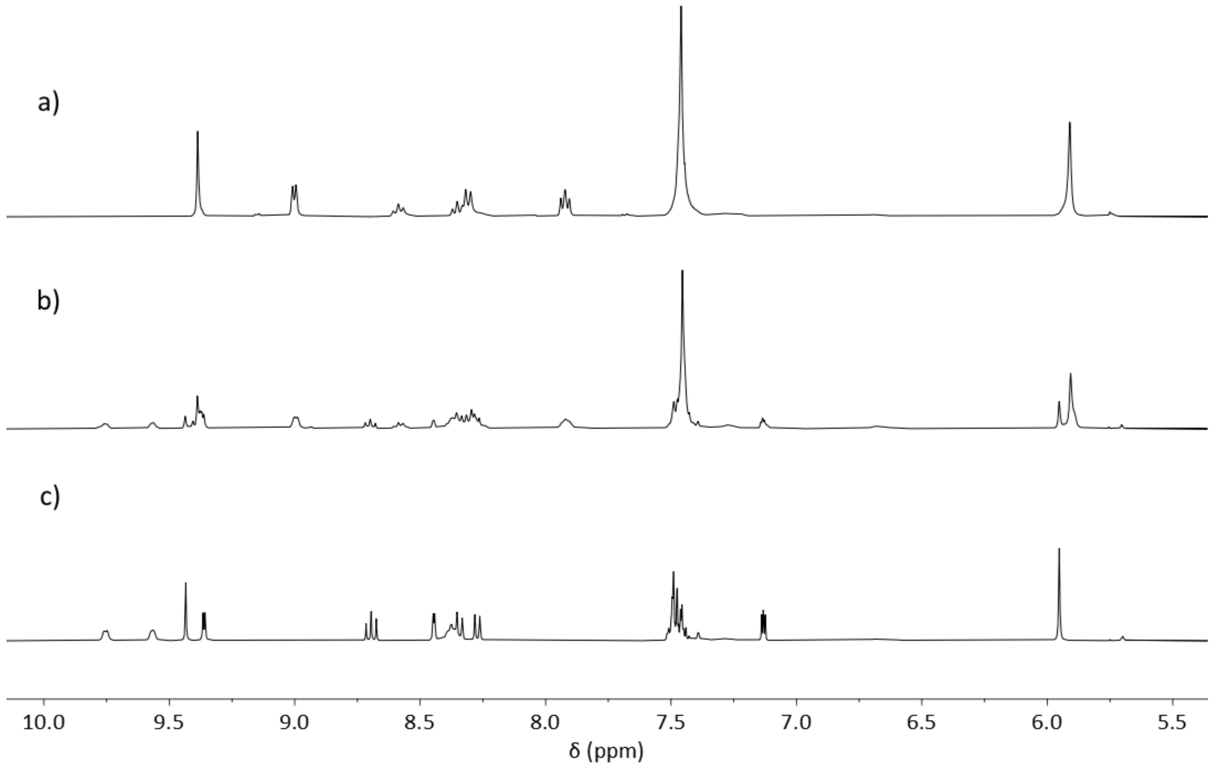

Figure 2.10 Stacked partial ^{1}H NMR spectra (400 MHz, [D$_6$]DMSO, 298 K) of a) $3_{\mathbf{DD}}\mathbf{1}_{\mathbf{AA}}$, b) $3_{\mathbf{DD}}\mathbf{1}_{\mathbf{AA}}$ and $3_{\mathbf{AD}}\mathbf{1}_{\mathbf{DA}}$, and c) $3_{\mathbf{AD}}\mathbf{1}_{\mathbf{DA}}$.

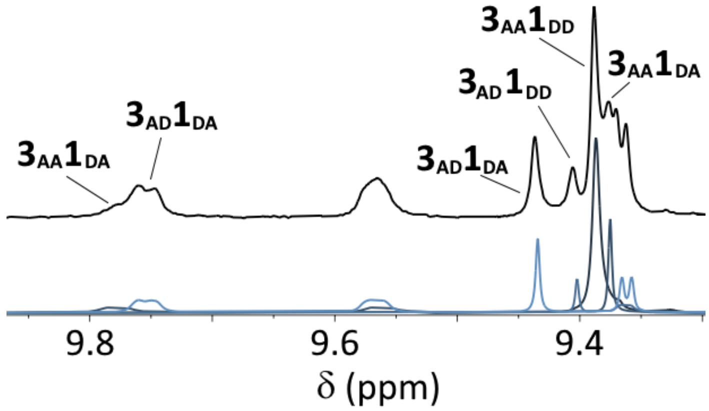

Figure 2.11 ^{1}H NMR spectra (400 MHz, [D$_6$]DMSO, 298 K) of the equilibrium mixture obtained from the 1:1 combination of $\mathbf{3}_{\mathbf{AA}}\mathbf{1}_{\mathbf{DD}}$ and $\mathbf{3}_{\mathbf{AD}}\mathbf{1}_{\mathbf{DA}}$ (*top*), and overlaid spectra of $\mathbf{3}_{\mathbf{AA}}\mathbf{1}_{\mathbf{DD}}$ (black), $3_{\mathbf{AD}}\mathbf{1}_{\mathbf{DA}}$ (blue), $\mathbf{3}_{\mathbf{AA}}\mathbf{1}_{\mathbf{DA}}$ (charcoal) and $\mathbf{3}_{\mathbf{AD}}\mathbf{1}_{\mathbf{DD}}$ (grey) with relative peak intensity set to 2:1 between 'unscrambled' and 'scrambled' species.

2.2. Quadruple complex combinations

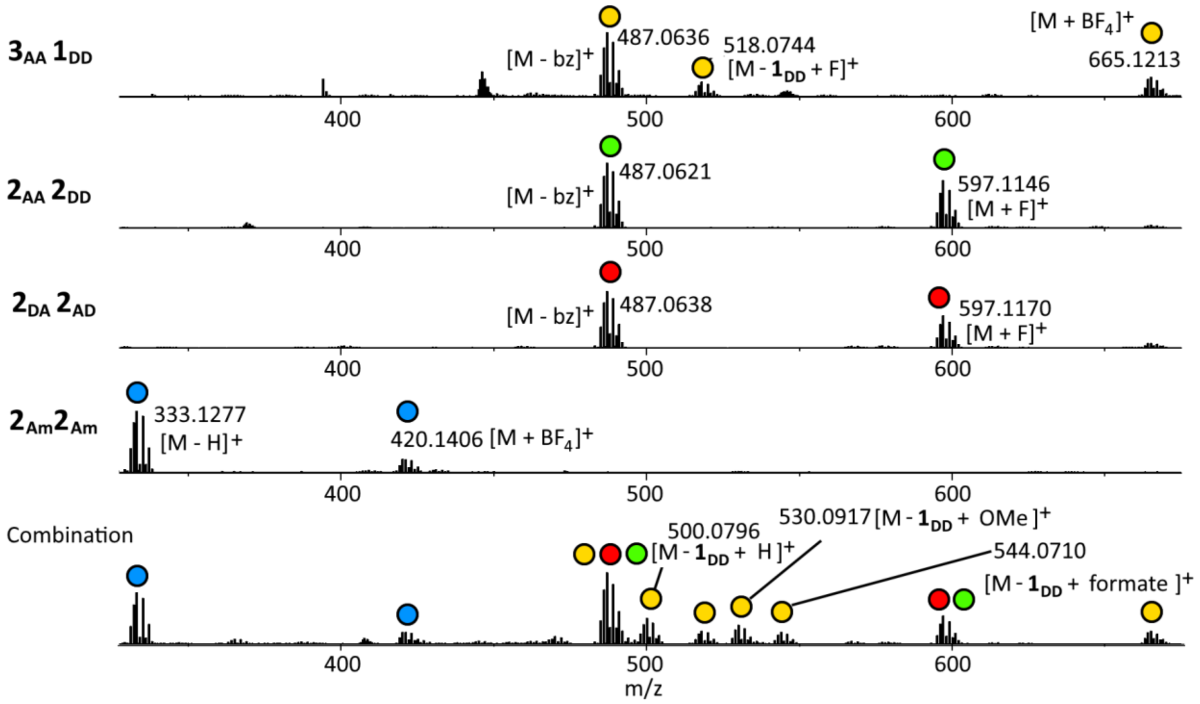

Figure 2.12 Partial mass spectra (DMSO/acetonitrile) for individual complexes $\mathbf{3}_{AA}\mathbf{1}_{DD}$, $\mathbf{2}_{AA}\mathbf{2}_{DD}$, $\mathbf{2}_{DA}\mathbf{2}_{AD}$, and $\mathbf{2}_{Am}\mathbf{2}_{Am}$ and their combination.

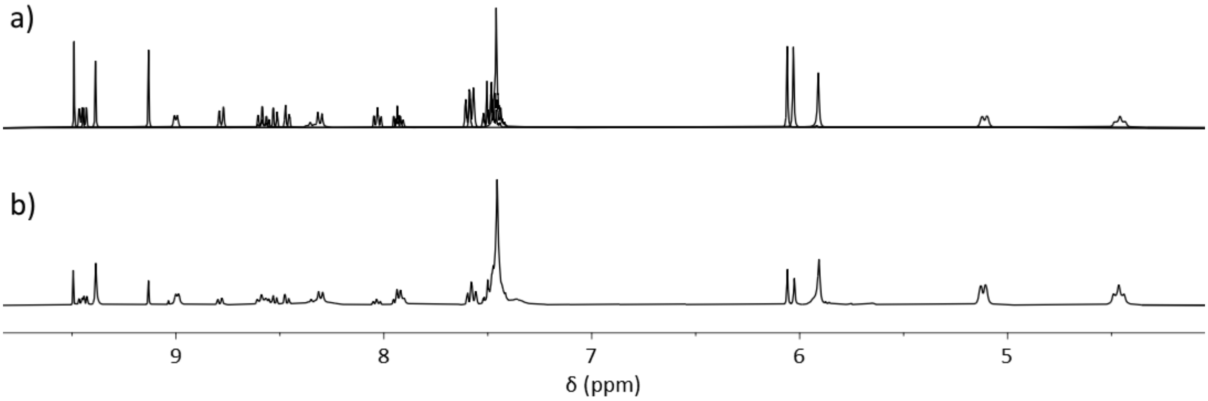

Figure 2.13 Partial ^{1}H NMR spectra (400 MHz, [D$_6$]DMSO, 298 K) spectra: a) overlaid four spectra of individual complexes $\mathbf{3}_{AA}\mathbf{1}_{DD}$, $\mathbf{2}_{AA}\mathbf{2}_{DD}$, $\mathbf{2}_{DA}\mathbf{2}_{AD}$, and $\mathbf{2}_{Am}\mathbf{2}_{Am}$ and b) the spectrum of their combination.

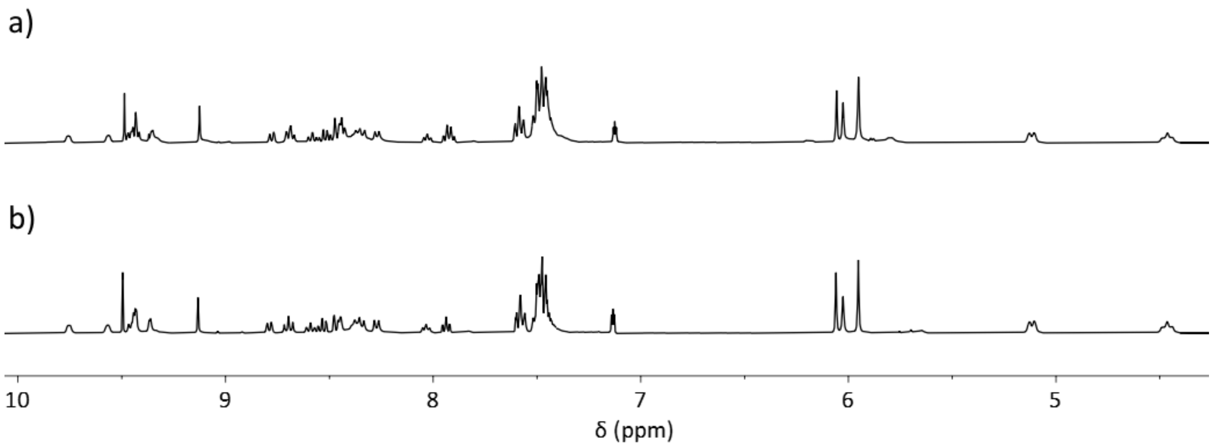

Figure 2.14 Partial ^{1}H NMR spectra (400 MHz, [D$_6$]DMSO, 298 K) spectra of a) the spectrum obtained from combining ligands $\mathbf{3_{AA}}$, $\mathbf{1_{DD}}$, $\mathbf{2_{AA}}$, $\mathbf{2_{DD}}$, $\mathbf{2_{DA}}$, and $\mathbf{2_{Am}}$ in a 1:1:1:1:2:2 ratio then adding 4 eq. Pd(II), and b) the combination of complexes $\mathbf{3_{AA}1_{DD}}$, $\mathbf{2_{AA}2_{DD}}$, $\mathbf{2_{DA}2_{AD}}$, and $\mathbf{2_{Am}2_{Am}}$.

3. Computations

DFT calculations were performed using the ORCA program version 4.0.[9] Structures were fully optimized using the BP86[10] functional with a def2-TZVP[11] basis set. Calculations were performed in a polarizable continuum solvent using a DMSO solvent field CPCM model. SCF iterations were considered converged when the energy change was less than 1×10^{-8} a.u. The geometry was considered optimized when the following tolerances were met: maximum gradient = 3×10^{-4} a.u., RMS gradient = 1×10^{-4} a.u., maximum displacement = 4×10^{-3} a.u., RMS displacement = 2×10^{-3} a.u.. Optimised structures are available as xyz files.

4. References

[1] A. Kirschning, C. Altwicker, G. Dräger, J. Harders, N. Hoffmann, U. Hoffmann, H. Schönfeld, W. Solodenko, U. Kunz, *Angew. Chem., Int. Ed.* **2001**, *40*, 3995-3998.

[2] T. Li, L. Guo, Y. Zhang, J. Wang, Z. Li, L. Lin, Z. Zhang, L. Li, J. Lin, W. Zhao, J. Li, P. G. Wang, *Carbohydrate Research* **2011**, *346*, 1083-1092.

[3] U. Monkowius, S. Ritter, B. König, M. Zabel, H. Yersin, *European Journal of Inorganic Chemistry* **2007**, *2007*, 4597-4606.

[4] K. J. Kilpin, E. L. Gavey, C. J. McAdam, C. B. Anderson, S. J. Lind, C. C. Keep, K. C. Gordon, J. D. Crowley, *Inorganic Chemistry* **2011**, *50*, 6334-6346.

[5] P. Danielraj, B. Varghese, S. Sankararaman, *Acta Crystallographica Section C* **2010**, *66*, m366-m370.

[6] in *CrysAlisPro*, Agilent Technologies, Yarnton, England, **2012**.

[7] G. M. Sheldrick, *Acta Crystallogr., Sect. A: Found. Crystallogr.* **2008**, *64*, 112-122.

[8] O. V. Dolomanov, L. J. Bourhis, R. J. Gildea, J. A. K. Howard, H. Puschmann, *J. Appl. Crystallogr.* **2009**, *42*, 339-341.

[9] F. Neese, *WIREs Computational Molecular Science* **2012**, *2*, 73-78.

[10] aA. D. Becke, *Physical Review A* **1988**, *38*, 3098-3100; bJ. P. Perdew, *Physical Review B* **1986**, *33*, 8822-8824; cJ. P. Perdew, W. Yue, *Physical Review B* **1986**, *33*, 8800-8802.

[11] A. Schäfer, H. Horn, R. Ahlrichs, *The Journal of Chemical Physics* **1992**, *97*, 2571-2577.
